# ATRP with ppb Concentrations of Photocatalysts

**DOI:** 10.1021/jacs.4c09927

**Published:** 2024-10-10

**Authors:** Halil Ibrahim Coskun, Ferdinando De Luca Bossa, Xiaolei Hu, Steffen Jockusch, Julian Sobieski, Gorkem Yilmaz, Krzysztof Matyjaszewski

**Affiliations:** † Department of Chemistry, Carnegie Mellon University, 4400 Fifth Avenue, Pittsburgh, Pennsylvania 15213, United States; ‡ Department of Chemistry and Center for Photochemical Sciences, Bowling Green State University, Bowling Green, Ohio 43403, United States

## Abstract

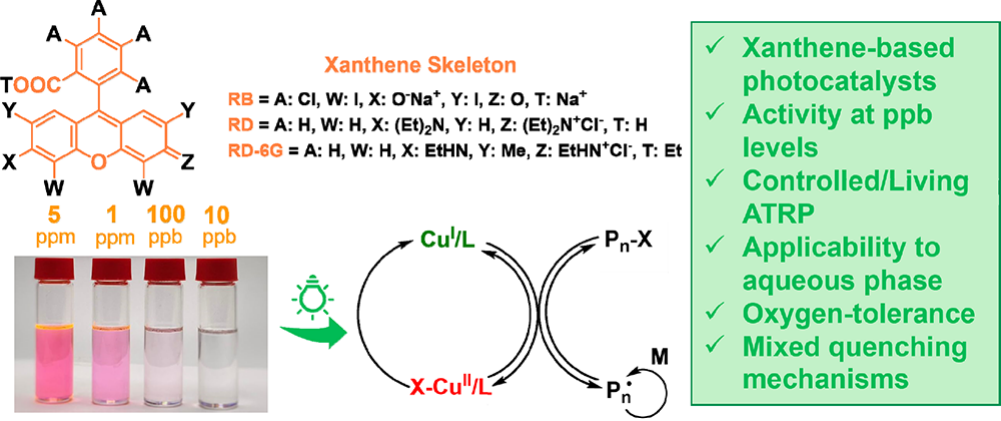

In atom transfer radical polymerization (ATRP), dormant alkyl halides are intermittently activated to form growing radicals in the presence of a Cu^I^/L/X-Cu^II^/L (activator/deactivator) catalytic system. Recently developed very active copper complexes could decrease the catalyst concentration to ppm level. However, unavoidable radical termination results in irreversible oxidation of the activator to the deactivator species, leading to limited monomer conversions. Therefore, successful ATRP at a low catalyst loading requires continuous regeneration of the activators. Such a regenerative ATRP can be performed with various reducing agents under milder reaction conditions and with catalyst concentrations diminished in comparison to conventional ATRP. Photoinduced ATRP (PhotoATRP) is one of the most efficient methods of activator regeneration. It initially employed UV irradiation to reduce the air-stable excited X-Cu^II^/L deactivators to the activators in the presence of sacrificial electron donors. Photocatalysts (PCs) can be excited after absorbing light at longer wavelengths and, due to their favorable redox potentials, can reduce X-Cu^II^/L to Cu^I^/L. Herein, we present the application of three commercially available xanthene dyes as ATRP PCs: rose bengal (RB), rhodamine B (RD), and rhodamine 6G (RD-6G). Even at very low Cu catalyst concentrations (50 ppm), they successfully controlled PhotoATRP. Well-defined polymers with preserved livingness were prepared under green LED irradiation, with subppm concentrations ([PC] ≥ 10 ppb) of RB and RD-6G or 5 ppm of RD. Interestingly, these PCs efficiently controlled ATRP at wavelengths longer than their absorption maxima but required higher loadings. Polymerizations proceeded with high initiation efficiencies, yielding polymers with narrow molecular weight distributions and high chain-end fidelity. UV–vis, fluorescence, and laser flash photolysis studies helped to elucidate the mechanism of the processes involved in the dual-catalytic systems, comprising parts per million of Cu complexes and parts per billion of PCs.

## Introduction

**Figure 1:**
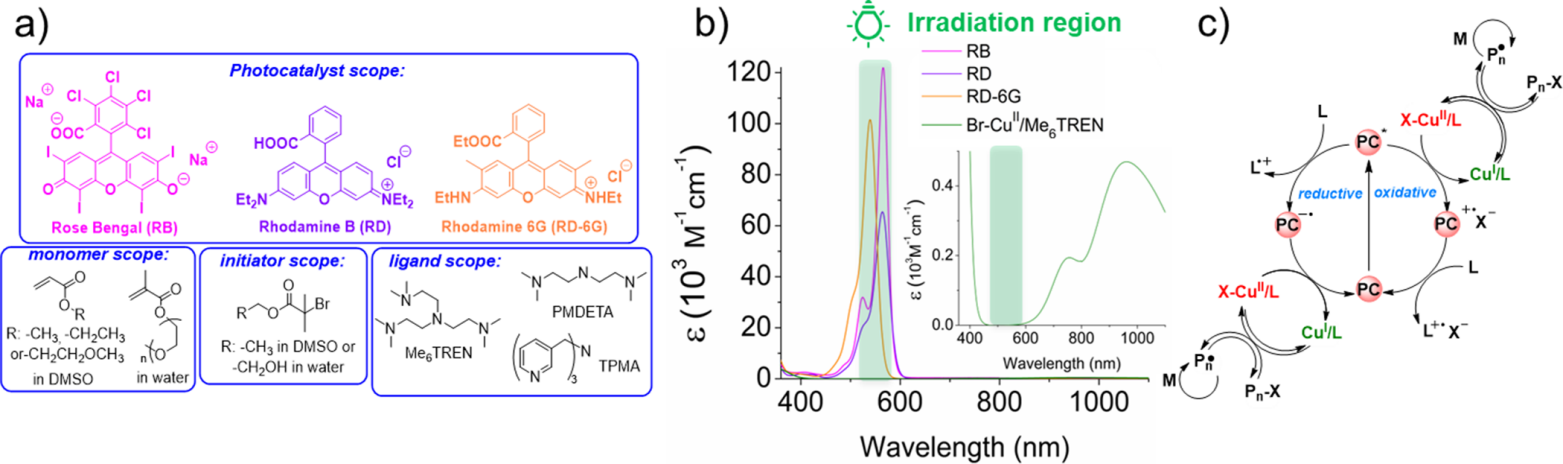
(a) Scope of photocatalysts (PCs), monomers, initiators, and ligands used in this study. (b) Comparison of the UV–vis spectra of PCs and CuBr_2_/Me_6_TREN in DMSO and the irradiation window of the applied green LED (inset magnified for CuBr_2_/Me_6_TREN). (c) Dual-catalysis PhotoATRP by oxidative and reductive quenching of a PC.

Atom transfer radical polymerization (ATRP) is a reversible deactivation radical polymerization (RDRP) process^1^ involving an intermittent exchange between growing radicals (P_*n*_^•^) and dormant alkyl halides (P_*n*_-X). This process is catalyzed by a redox-active catalytic system consisting of an activator in a lower oxidation state and a halide-bearing deactivator in a higher oxidation state (Figure 1c, Cu^I^/L, and X-Cu^II^/L, respectively). Subsequently, P_*n*_^•^ adds monomer units and is deactivated by X-Cu^II^/L to restore the halogen-capped dormant species and Cu^I^/L.^2,3^ In this manner, ATRP enables the synthesis of tailor-made polymers with controlled molecular weight and low dispersity, especially if deactivation is rapid relative to propagation.^4−6^

The ATRP equilibrium dictates the fraction of propagating radicals and is affected by the structure of the dormant species and catalyst’s ligand, L. However, even the recently developed (super)active catalysts cannot be used at the ppm level on their own.^7−10^ Unavoidable radical termination results in the irreversible oxidation of activators to deactivator species, halting ATRP and limiting monomer conversions. Therefore, successful ATRP at very low catalyst loadings required continuous regeneration of the activators.^11,12^ Such regenerative ATRP techniques include activators regenerated by electron transfer (ARGET),^13,14^ initiators for continuous activator regeneration (ICAR),^15^ supplemental activator and reducing agent (SARA) ATRP,^16,17^ electrochemically mediated ATRP (eATRP),^18^ mechanically driven ATRP,^19−22^ and photoinduced ATRP (PhotoATRP).^23−27^

Light is a powerful tool^28^ used in conventional radical,^29^ cationic,^30,31^ step-growth,^32^ and controlled radical polymerization techniques, including ATRP^1,33−38^ and reversible addition–fragmentation-transfer (RAFT) polymerization.^39−43^ The associated low/tunable energy requirements, affordable oxygen tolerance, and spatiotemporal control provide advantages for 3D printing, lithography, holograms, coatings, varnishes, composites, and medicine.^44−51^

Initially, PhotoATRP required UV irradiation to form excited-state deactivators ([X-Cu^II^/L]*), which were readily reduced in the presence of sacrificial electron donors (typically amines or excess ligands).^52^ However, the drawbacks of using UV light include poor light penetration and potential triggering of side reactions. This is especially relevant in biologically related systems due to diminished damage of cells or tissues and longer penetration of green or red light.^53−55^

Recently, considerable efforts have been made to understand the photoactivity of ATRP systems^56^ and expand the associated spectral range by using organic photocatalysts (PCs) to regenerate Cu^I^/L activators in a dual-catalysis approach.^57−60^ Important features include operability in organic and aqueous media,^61,62^ oxygen tolerance,^63^ and control over polymerization under minimal PC loadings.^64−67^ In a recent example, dual-catalysis PhotoATRP was performed with methylene blue as a PC and Br–Cu^II^/Me_6_TREN under red and near-infrared light.^68^ The dual-catalytic system operated in homogeneous organic and aqueous media and emulsion systems.^69^ The associated oxygen tolerance allowed for high-throughput screening at reduced reaction volumes for facile process optimization.

Herein, we report dual-catalytic PhotoATRP used at very low concentrations (ppm and even ppb levels) using three commercially available xanthene dyes (Figure 1a): rose bengal (RB), rhodamine B (RD), and rhodamine 6G (RD-6G). Well-controlled polymerization was afforded in both organic (methyl acrylate in DMSO) and aqueous (oligo(ethylene oxide) methacrylate, OEOMA, *M*_n_ ∼ 500) media. Green and red LEDs were used as the irradiation sources, and the polymerizations were operated under either a nitrogen atmosphere or ambient/open-air conditions. The associated kinetics, evolution of molecular weights and dispersities, temporal control, and chain-end functionality were investigated. Spectroscopic studies were performed to clarify the mechanism of the processes involved in this dual-catalytic system.

## Results and Discussion

The UV–vis spectra of the PCs were recorded in DMSO (Figure 1b). RB and RD displayed maximum absorptions at λ_max_ = 565 nm, while RD-6G displayed maximum absorptions at λ_max_ = 540 nm. Br–Cu^II^/Me_6_TREN is transparent in the λ ∼ 400–600 nm region, permitting polymerizations under green and red-light irradiation without interference (Figure 1b). Depending on the photochemical properties of the xanthene dyes, the excited state of photocatalysts (PC*) can be quenched through oxidative or reductive mechanisms (Figure 1c). The former case involves a single electron transfer from PC* to X-Cu^II^/L, thereby oxidizing PC to the radical cation PC^•+^(X^–^) and regenerating Cu^I^/L as reported for Eosin Y.^35^ In the latter path, PC* accepts an electron from sacrificial electron donors (e.g., ligand, L), forming radical anions (PC^•–^) that reduce X-Cu^II^/L to Cu^I^/L (residual halide forms an ion pair, L^•+^X^–^), as observed for methylene blue.^68^ Therefore, fluorescence and laser flash photolysis were used to determine the mechanism of these reactions with the investigated xanthene dyes. In all cases, the Cu-ATRP redox pair (Cu^I^/L, X-Cu^II^/L) controls the activation and deactivation of chain ends and thus the molecular weights and dispersities of the resulting polymers. At the same time, PCs are responsible for the regeneration of Cu^I^/L activators, which affects the polymerization rate.

### Photoinduced Polymerizations and Kinetics

**Figure 2:**
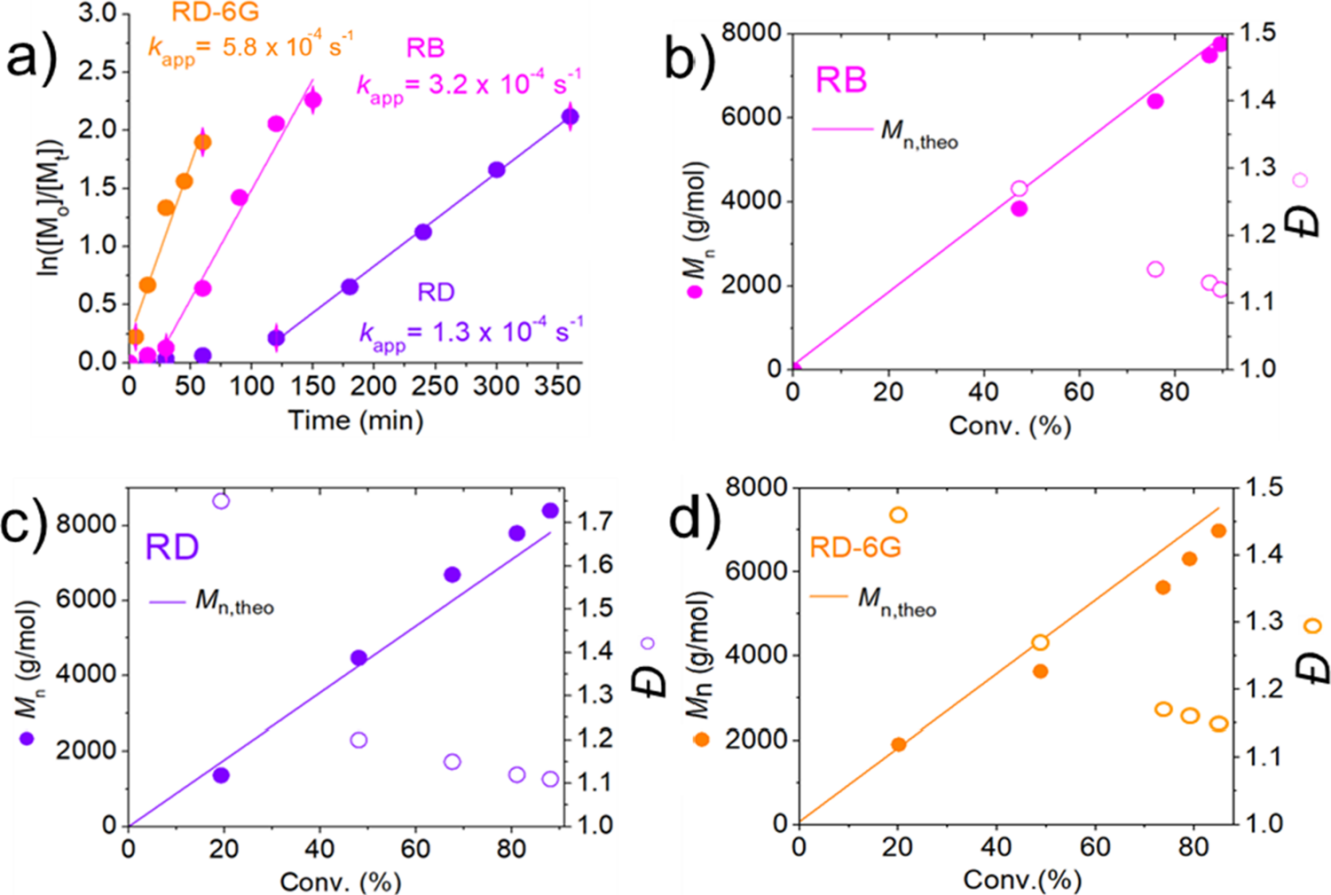
(a) Kinetics of MA polymerization using different PCs under the same reaction conditions ([MA]/[EBiB]/[CuBr_2_]/[Me_6_TREN]/[PC] = 100/1/0.005/0.015/0.0005, [PC] = 5 ppm, [CuBr_2_] = 50 ppm, V_MA_/V_DMSO_: 1/1, λ ∼ 525 nm, under a N_2_ atmosphere). (b–d) Plots of *M*_n_ vs Conv. (%) for different PCs.

The kinetics of MA polymerization by ATRP were monitored using a Schlenk flask under a N_2_ atmosphere with molar ratios: ([MA]/[EBiB]/[CuBr_2_]/[Me_6_TREN]/[PC]: 100/1/0.005/0.015/0.0005, [PC] = 5 ppm, V_MA_/V_DMSO_: 1/1) and exposed to green LED irradiation at 525 nm. Aliquot samples were periodically taken from the reaction mixture at defined intervals and analyzed by ^1^H NMR and GPC. ATRP with the RB and RD showed induction periods. No induction period was observed for RD-6G, suggesting rapid activator generation (see Mechanistic Investigations). Semilogarithmic kinetic plots were linear, confirming the constant concentration of growing radicals (Figure 2a). Linear first-order kinetic plots were also observed with 100 ppb RD-6G, confirming the efficient ATRP under subppm PC concentrations (Figure S1). The apparent rate constants, derived from the kinetic slopes, followed the order RD-6G > RB > RD, in agreement with the polymerization results collected in Table 1 (GPC traces are shown in Figure S2). The determined molecular weights (*M*_n,GPC_) increased with conversion for all of the PCs, while the dispersity values decreased with conversion, as is typical for a regenerative ATRP.

**Table 1:** PhotoATRP of MA Using EBiB, CuBr_2,_ and Me_6_TREN under Different Conditions^a^

PC	entry	[CuBr_2_]/[Me_6_TREN]/[PC]	[PC] (ppb)	[CuBr_2_] (ppm)	time (h)	conv. (%)^b^	*M*_n,theo_ (g·mol^–1^)^c^	*M*_n,GPC_ (g·mol^–1^)^d^	*Đ*^d^	*I*^e^
	1	0.005/0.015/0	0	50	9	<5				
RB	2	0.005/0.05/0.0005	5000	50	3	94	8280	7800	1.14	1.06
	3	0.005/0.015/0.0005	5000	50	2.5	90	7940	7750	1.12	1.02
	4	0.005/0.015/0.0001	1000	50	3	91	8020	9400	1.14	0.85
	5	0.001/0.003/0.0001	1000	10	3	57	5100	5000	1.73	1.02
	6	0.005/0.015/0.00001	100	50	9	84	7420	8400	1.17	0.88
	7	0.005/0.015/0.000005	50	50	24	86	7400	8000	1.16	0.92
	8	0.005/0.015/0.000001	10	50	9	30	2780	4000	1.40	0.70
	9	0.005/0.015/0.000001	10	50	24	54	4850	5400	1.26	0.90
RD	10	0.005/0.05/0.0005	5000	50	4	97	8540	7750	1.16	1.10
	11	0.005/0.015/0.0005	5000	50	6	88	7760	8400	1.11	0.92
	12	0.002/0.02/0.0002	2000	20	4					
RD-6G	13	0.005/0.015/0.0005	5000	50	1.5	86	7600	7000	1.15	1.08
	14	0.005/0.015/0.0001	1000	50	1.5	82	7250	7900	1.16	0.92
	15	0.005/0.015/0.00001	100	50	1.5	87	7700	9100	1.11	0.85
	16	0.001/0.003/0.00001	100	10	1.5	39	3550	3450	1.88	1.03
	17	0.005/0.015/0.000005	50	50	1.5	68	6050	6700	1.18	0.90
	18	0.005/0.015/0.000001	10	50	6	58	5180	6400	1.23	0.81

a [MA]/[EBiB]/[CuBr_2_]/[Me_6_TREN]/[PC]: 100/1/*x*/*y*/*z*, V_MA_/V_DMSO_ = 1/1, under N_2_ (λ ∼ 525 nm, intensity: 25 mW·cm^–2^).

b Determined by ^1^H NMR.

c *M*_n,theo_ = conv. (%) × 86 + 195.

d Determined by GPC in THF using poly(methyl methacrylate) standards.

e Initiation efficiency (*I*) = *M*_n,theo_/*M*_n,GPC_.

*M*_n,GPC_ values agreed with calculated *M*_n,theo_ values, indicating high initiation efficiencies for all employed PCs (Figure 2b–d). Polymers with monomodal molecular weight distributions were obtained, even at concentrations of PCs as low as 5 ppm. No significant polymerization was observed without PCs, indicating that green light alone cannot appreciably (re)generate Cu^I^/L activators (entry 1). When PC concentrations decreased below 5 ppm, RD was inefficient (entry 12). In contrast, RB and RD-6G effectively provided fast and controlled polymerizations even at 1 ppm loadings (entry 4: 3 h for RB and entry 14: 1.5 h for RD-6G). Longer irradiation was required with the PC concentrations at ppb levels. At 100 ppb RB, the reaction reached 84% conversion in 9 h and yielded polymers with low dispersity (entry 6, *Đ* = 1.17). A similar conversion (86%) was reached in 24 h when 50 ppb RB was used without compromising molecular weight control (entry 7, *Đ* = 1.16). At 10 ppb RB loadings, a 30% conversion was attained in 9 h, giving polymers with higher dispersity (entry 8, *Đ* = 1.40). When irradiation time was increased to 24 h, the conversion reached 54%, yielding polymers with narrower molecular weight distributions (entry 9, *Đ* = 1.26). Systems with RD-6G reached similar conversions in 1.5 h, even when the PC concentration was reduced from 1 ppm (82%) to 100 ppb (87%) while also maintaining control (entries 14 and 15). When 50 ppb of RD-6G was used, polymerization reached 68% conversion with good control (entry 17, *Đ* = 1.18). With 10 ppb RD-6G, the conversion decreased to 58% in 6 h with an acceptable molecular weight control (entry 18, *Đ* = 1.23). The initiation efficiencies (*I*) were close to unity for all PCs.

The optimal concentration of [CuBr_2_]_0_ was ca. 50 ppm in all cases. The [CuBr_2_]/[L] ratio was maintained at 1/3, as similar results were obtained at a 1/10 ratio (entries 2 and 3). When the Cu^II^ loading was reduced to 10 ppm, the dispersities increased (entries 5 and 16) for RB and RD-6G because the control in ATRP is defined by the deactivation rate with Br–Cu^II^/L.^6^ A higher dispersity could also be related to lower conversion (lower DP).^70^ Experiments with PMDETA (Table S1 and Figure S3) required a higher catalyst loading and longer irradiation to achieve similar (nearly quantitative) conversions. This relates to a much higher *K*_ATRP_ for Cu^I^/Me_6_TREN than for Cu^I^/PMDETA, which remains mostly in the deactivator state.^71,72^ Hence, Cu/Me_6_TREN affords both faster reduction and deactivation during the ATRP steady state at low catalyst loadings.^70^

The effect of [PC], [CuBr_2_], and [free Me_6_TREN] on the polymerization rate was investigated using RD-6G as an example (Figure S4). When the [RD-6G] concentration was reduced to subppm levels, the rate was slightly decreased, suggesting that the polymerizations could be conducted using such low concentrations. The [free Me_6_TREN] increased the polymerization rate, but no consistent effect was observed when [CuBr_2_] was changed. This might be related to the loss of control at lower CuBr_2_ loadings, which switched the system to an uncontrolled radical polymerization (see Table 1, entry 16, *Đ* = 1.88).

The dual-catalytic system was applied to other acrylic monomers, such as ethyl methacrylate and methoxyethyl acrylate with 50 ppm of Br–Cu^II^/Me_6_TREN and RD (5 ppm) and 100 ppb of RB and RD-6G. High conversions were reached even at subppm PC loadings. Polymerizations had high initiation efficiencies (*I* ≤ 0.98), affording polymers with low dispersity (*Đ* ≤ 1.18) (Table S2 and Figure S5).

The efficacy of the systems was tested for various targeted degrees of polymerization under identical PC concentrations (5 ppm, Table S3, and DP_target_ up to 1600). Remarkably, the *I* values were 0.94 even for DP_target_ = 1600, illustrating the exceptional efficiency of RB and RD-6G. RD did not lead to a precipitable polymer at DP_target_ = 1600. Notably, the molecular weight distributions were narrow (*Đ* ≤ 1.08) even when higher molecular weights were targeted (Figure S6). The system was also efficient at 100 ppb RB and RD-6G loadings, as reflected by polymers with low dispersities (*Đ* = 1.03–1.08) and high *I* values (*I* ≥ 0.87) (Figure S7).

### Irradiation at Longer Wavelengths

Efficient photochemical reactions are possible when PCs sufficiently absorb light. However, a recent study revealed that photochemical reactivity may not match absorptivity.^73^ Therefore, we investigated the efficiency of PCs in PhotoATRP at wavelengths beyond their maximum absorption band. The UV–vis spectra of the PCs recorded at higher concentrations exhibited tail absorptions at longer wavelengths (Figure S8). The PhotoATRP results with PCs under red LED irradiation (λ = 640 nm) are shown in Table 2. All PCs required higher concentrations (50 instead of 5 ppm) to achieve high conversions under red-light irradiation.

**Table 2:** PhotoATRP of MA at Different Wavelengths and PC Concentrations^a^

PC	[PC] (ppm)	λ (nm)	conv. (%)^b^	*M*_n,theo_^c^	*M*_n,GPC_^d^	*Đ*^d^
RB	5^e^	525	90	7950	7800	1.12
	5	640				
	50	640	88	7750	10,000	1.05
RD	5^e^	525	88	7750	8400	1.11
	5	640				
	50	640	57	5100	6300	1.05
RD-6G	5^e^	525	86	7600	7000	1.18
	5	640				
	50	640	92	8100	6750	1.09

a [MA]/[EBiB]/[CuBr_2_]/[Me_6_TREN]/[PC]: 100/1/0.005/0.015/*x*, [CuBr_2_] = 50 ppm, V_MA_/V_DMSO_ = 1/1, under N_2_, intensity: 25 mW·cm^–2^, reaction times: 3 h for RB, 6 h for RD, and 1.5 h for RD-6G.

b Determined by ^1^H NMR.

c *M*_n,theo_ = conv. (%) × 86 + 195.

d Determined by GPC in THF using poly(methyl methacrylate) standards.

e Data taken from Table 1.

### Temporal Control and Chain-End Fidelity

**Figure 3:**
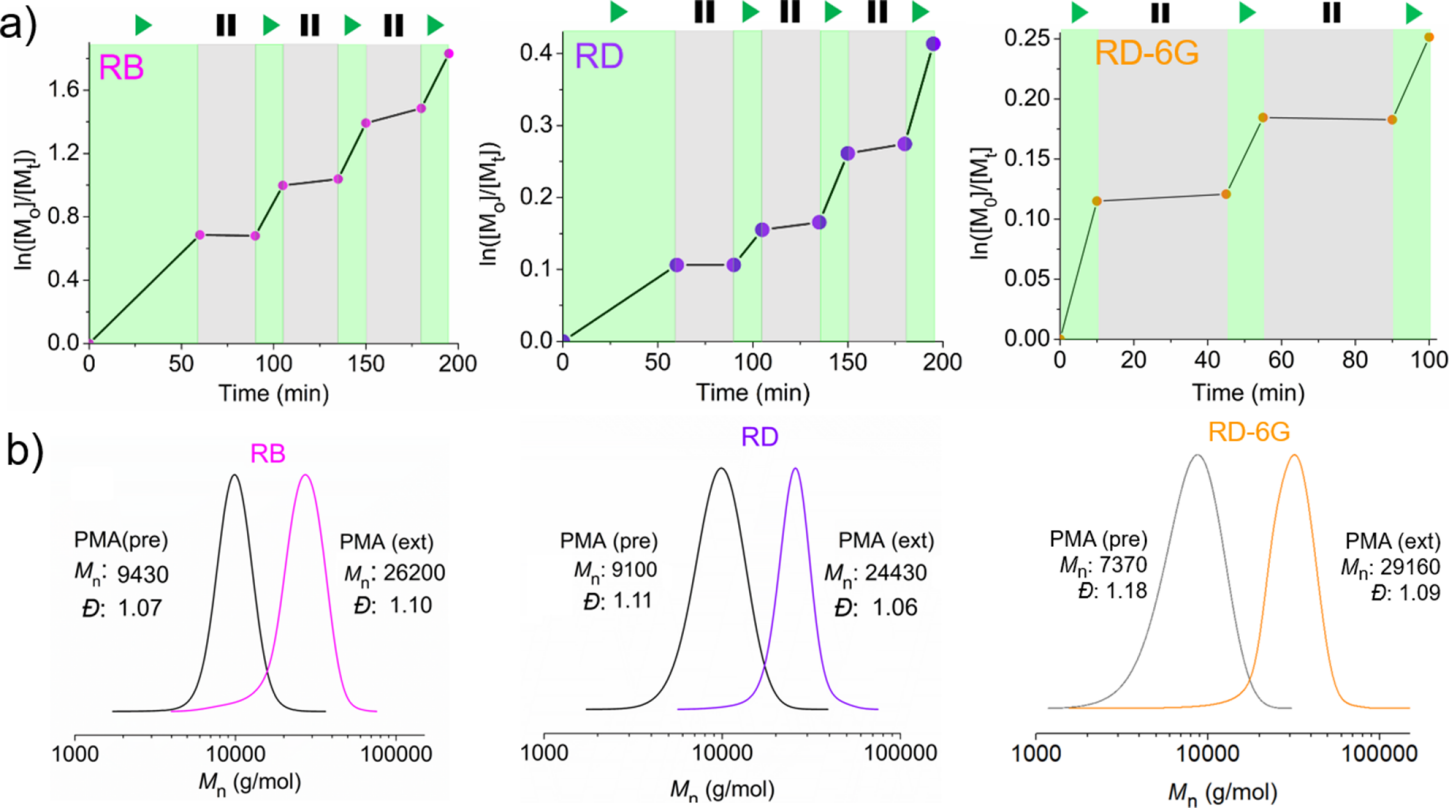
(a) Temporal control results for [MA]/[EBiB]/[CuBr_2_]/[Me_6_TREN]/[RB or RD] = 100/1/0.005/0.015/0.0005, [CuBr_2_] = 50 ppm, [RB or RD] = 5 ppm, and [MA]/[EBiB]/[CuBr_2_]/[Me_6_TREN]/[RD-6G] = 100/1/0.002/0.006/0.0002, [CuBr_2_] = 20 ppm, [RD-6G] = 2 ppm, V_MA_/V_DMSO_ = 1/1, under N_2_, green LED (λ ∼ 525 nm, intensity: 25 mW·cm^–2^). Green grids represent lights switched on; gray grids represent dark periods. (b) Comparisons of the GPC traces of precursor PMA (PMA(pre)) and chain-extended PMA (PMA(ext)) ([MA]/[PMA(pre)]/[CuBr_2_]/[Me_6_TREN]/[PC] = 300/1/0.015/0.045/0.0015, [CuBr_2_] = 50 ppm, [PC] = 5 ppm, V_MA_/V_DMSO_ = 1/1, under N_2_, green LED (λ ∼ 525 nm, intensity: 25 mW·cm^–2^).

Temporal control was investigated for all PCs by applying sequences of light on/off cycles (Figure 3a). All PCs showed excellent temporal control under similar conditions since no significant conversion was observed during the dark periods, confirming the key role of PCs in regenerating Cu^I^/L activators. The temporal control on PhotoATRP was demonstrated for PCs at the ppb level using RD-6G (100 ppb, Figure S9a) as an example.

Subsequent chain extensions of the macroinitiators synthesized with 5 ppm of PCs confirmed their chain-end fidelity. GPC analyses showed clear shifts to higher molecular weights, demonstrating successful functional group control for all employed PCs (Figure 3b). The chain-end functionality was preserved even with 100 ppb of RB and RD-6G, as confirmed by successful chain extension experiments (Figure S9b).

### ATRP in an Aqueous Medium in Open Air

The performance of the PCs in aqueous media and under air was tested (Table 3). Oligo(ethylene oxide) methyl ether methacrylate (OEOMA_500_) was polymerized by employing the PCs with the 2-hydroxyethyl α-bromoisobutyrate (HOBiB) initiator and Br–Cu^II^/TPMA in 1 × PBS with DMSO (10%, v/v) under green LED irradiation (λ ∼ 525 nm, intensity: 25 mW·cm^–2^). TPMA was preferred over Me_6_TREN because Br–Cu^II^/Me_6_TREN disproportionates in aqueous media.^74^ The polymerizations were performed in open vials without nitrogen bubbling. RB showed remarkable photocatalytic activity, achieving quantitative conversion even in open air, demonstrating efficient oxygen tolerance. Under the same conditions, with RD and RD-6G, lower conversions were achieved (63 and 65%, respectively) due to partial oxygen retardation. However, dispersity values remained low (*Đ*: 1.16–1.19), and initiation efficiencies were close to unity (*I*: 0.97–0.99). This suggested that the excited-state PCs facilitated oxygen scrubbing, preventing inhibition or severe retardation of the polymerization.

**Table 3:** Comparison of PhotoATRP of OEOMA_500_ Using HOBiB and Br-CuBr/Me_6_TREN in an Aqueous Medium in Open Air^a^

PC	conv. (%)^b^	*M*_n,theo_^c^	*M*_n,MALS_^d^	*Đ*^d^	*I*^e^
RB	97	97,000	99,700	1.19	0.97
RD	63	63,000	63,700	1.17	0.99
RD-6G	65	65,000	65,600	1.16	0.99

a [OEOMA]/[HOBiB]/[CuBr_2_]/[TPMA]/[PC] = 200/1/0.3/0.9/0.025, [PC] = 125 ppm, [CuBr_2_] = 1500 ppm, V_MA_/V_PBS_: 1/1, 10% DMSO by volume, λ ∼ 525 nm, intensity: 25 mW·cm^–2^, reaction time: 1 h.

b Determined by ^1^H NMR.

c *M*_n,theo_= conv. (%) × 500 + 211.

d Determined by GPC in DMF with a multiangle light scattering detector.

e (*I*) = *M*_n,theo_/*M*_n,GPC_.

### Contributions of Electron Transfer Processes

Calculations were made to predict the nature of the electron transfer processes. The redox process can occur between the ground states (S_0_), singlet states (S_1_), or triplet states (T_1_) of the PCs and the S_0_ of Br–Cu^II^/Me_6_TREN deactivator or the excess ligand (electron donor) because they are transparent at irradiation wavelengths (λ = 525 nm, Figure 1b).

The thermodynamic feasibility of a ground-state redox process can be calculated by using eqs 1 and 2, where *E*_ox_ and *E*_red_ represent oxidation and reduction potentials of the reactants, respectively. Δ*E* is the redox potential, Δ*G* is the Gibbs free energy of the redox reaction, *n* is the number of electrons transferred, and *F* is the Faraday constant (96.485 kJ·V^–1^·mol^–1^).
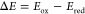
1
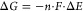
2

When electron transfer is facilitated by light, it involves the excited state of one of the components (PC* in this case). Therefore, the energy of the excited states (E*) should be considered. The formula for the calculation of Δ*E* is shown in eq 3:
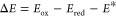
3

The corresponding Δ*G* values of the redox processes between the PCs and Br–Cu^II^/Me_6_TREN (*E*_red_ = −0.33 V (vs SCE in acetonitrile))^75^ or Me_6_TREN (*E*_ox_ = 0.51 V (vs SCE in acetonitrile)) are shown in Table 4. Accordingly, no redox reaction should occur involving the ground states of the PCs. However, both oxidative and reductive quenching mechanisms involving either the singlet or the triplet excited states of PCs are thermodynamically feasible, as shown in Table 4 (bold fonts, Δ*G* < 0 for S_1_ and T_1_).

**Table 4:** Photophysical Properties of the PCs and the Related Theoretical Energy Changes for Possible Electron Transfer Processes^a^

	excited-state energies (eV)		S_0_ ground-state redox potentials (V vs SCE)		S_1_ redox potentials (V vs SCE)		T_1_ redox potentials (V vs SCE)		Δ*G*_ox_ (e^–^ transfer to Br–Cu^II^/Me_6_TREN (kJ·mol^–1^))			Δ*G*_red_ (e^–^ transfer from Me_6_TREN (kJ·mol^–1^))		
PC	*E*^S1^_0.0_	*E*^T1^_0.0_	*E*^red^_1/2_	*E*^ox^_1/2_	*E*^S1^_red_	*E*^S1^_ox_	*E*^T1^_red_	*E*^T1^_ox_	S_0_	S_1_	T_1_	S_0_	S_1_	T_1_
RB^76^	2.17	1.8	–0.99	+0.84	+1.18	–1.33	+0.81	–0.96	112.9	**–96.5**	**–60.8**	144.7	**–64.6**	**–28.9**
RD^76^	2.22	1.80	–0.96	+0.91	+1.26	–1.31	+0.84	–0.89	119.6	**–94.5**	**–54.0**	141.8	**–72.4**	**–31.8**
RD-6G	2.32^77^	2.09^78^	–1.14^77^	+1.23^79^	+1.18^77^	–1.09^77^	+0.95^77^	–0.86^79^	150.5	**–73.3**	**–51.1**	159.2	**–64.6**	**–42.4**

a *E*_ox_(Me_6_TREN): 0.51 V (vs SCE in acetonitrile), *E*_red_(Br–Cu^II^/Me_6_TREN): −0.33 V (vs SCE in acetonitrile).^75^ All values for RB and RD were taken from ref (76) (in methanol, potentials originally reported relative to the Ag/AgCl reference electrode were referenced to SCE by subtracting 0.039 V from the reported values).^80,81^

### Mechanistic Investigations

The photophysical properties of the ground-state PCs in DMSO and water were analyzed by UV–vis spectroscopy (Figure S10). The molar absorption coefficients (ε) of RB and RD were very similar in water, but the ε value of RD was significantly lower in DMSO. This is in agreement with previous reports, which showed that RD exists in an “open” quinoid/lactone equilibrium.^82^ The equilibrium is shifted toward the colorless lactone form in organic solvents.

**Figure 4:**
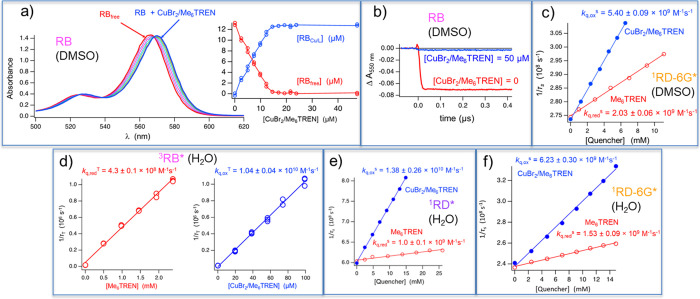
(a) Absorption spectra of RB (12.9 μM) in DMSO in the absence (red) and presence of various amounts of Br–Cu^II^/Me_6_TREN (1:1). Inset: plot of concentration of free RB and RB associated with Br–Cu^II^/Me_6_TREN vs concentration of Br–Cu^II^/Me_6_TREN derived from absorbance data. (b) Kinetic trace of transient absorbance of RB monitored at 550 nm in the absence (red) and presence of 50 μM Br–Cu^II^/Me_6_TREN (1:1) (blue) after pulsed laser excitation (λ_ex_ = 532 nm, 7 ns pulse width) in deoxygenated DMSO. (c) Determination of the bimolecular rate constants *k*_q_^S^ of quenching of RD-6G singlet excited states by Me_6_TREN (red) and Br–Cu^II^/Me_6_TREN (1:1) (blue) in DMSO by fluorescence lifetime measurements (λ_ex_ = 496 nm, pulsed LED). Plot of the inverse fluorescence lifetime of RD-6G determined by time-correlated single photon counting (λ_em_ = 568 nm) vs varying concentration of the quencher. (d) Determination of the bimolecular rate constants *k*_q_^T^ of the quenching of RB triplet states by Me_6_TREN (left) and Br–Cu^II^/Me_6_TREN (1:1) (right) in H_2_O was performed using laser flash photolysis (λ_ex_ = 532 nm, 7 ns pulse width, [RB] = 6.3 μM). Plot of the inverse triplet lifetime of RB (determined from the decay of the transient absorption at 620 nm) vs varying concentrations of Me_6_TREN (red) or Br–Cu^II^/Me_6_TREN (1:1) (blue). (e) Determination of the bimolecular rate constants *k*_q_^s^ of quenching of RD singlet excited states by Me_6_TREN (red) and Br–Cu^II^/Me_6_TREN (1:1) (blue) in H_2_O fluorescence lifetime measurements (λ_ex_ = 496 nm, pulsed LED). Plot of the inverse fluorescence lifetime of RD (determined by time-correlated single photon counting; λ_ex_ = 575 nm) vs varying concentration of the quencher. (f) Determination of the bimolecular rate constants *k*_q_^s^ of quenching of RD-6G singlet excited states by Me_6_TREN (red) and Br–Cu^II^/Me_6_TREN (1:1) (blue) in H_2_O fluorescence lifetime measurements (λ_ex_ = 496 nm, pulsed LED). Plot of the inverse fluorescence lifetime of RD-6G (determined by time-correlated single photon counting; λ_ex_ = 555 nm) vs varying concentration of the quencher.

Then, the UV–vis spectra of the PCs in the presence and absence of CuBr_2_ and/or Me_6_TREN in DMSO were measured. The absorption spectrum of RB displayed a red-shift (Δλ = 5 nm) in the presence of Br–Cu^II^/Me_6_TREN, likely due to ion pair formation in DMSO (Figure S11). To evaluate the stoichiometry of the complex, RB in DMSO was titrated with Br–Cu^II^/Me_6_TREN ([CuBr_2_]/[Me_6_TREN] = 1/1) (Figure 4a), showing an overall 1/1 molar ratio between RB and the Cu^II^-species (Figure 4a, inset). In deionized water, the maximum absorption remained at λ = 550 nm, indicating the dissociation of the ion pair (Figure S12). This red-shift was not observed in the case of RD, suggesting that the ionic interaction exists between the phenolate functionality in RB and the Cu^II^-complex.

The absorption coefficient was smaller in DMSO than expected (see comparison of absorption spectra in DMSO and water, Figure S10). The absorbance of RD was further quenched in the presence of Br–Cu^II^/Me_6_TREN ([RD]/[CuBr_2_]/[Me_6_TREN] = 1/10/10 and excess Me_6_TREN ([RD]/[CuBr_2_]/[Me_6_TREN] = 1/10/30, reaction stoichiometry) (Figure S13). This is possibly due to lactonization, a known phenomenon for pH-sensitive dyes.^83^ Assuming the absorption coefficients of RB and RD are the same (see absorption spectra of RB and RD in water, where no lactonization occurs), the fraction of the lactone form was estimated ca. 99% in DMSO (Figure S10). Therefore, for ATRP with 5 ppm RD (Table 1, entry 9), the concentration of the photoactive quinoid form of RD was ca. 50 ppb, similar to a total low ppb for RB and RD-6G. In contrast, RD-6G does not have a free carboxylic acid moiety that can lactonize, which makes it much more stable in a broad range of pH. Indeed, the UV spectrum of RD-6G remained unchanged in the presence of the Cu catalyst components (Figure S14).

Then, the UV–vis spectra of the PCs in the presence of the Cu catalyst components were analyzed in water. Notably, RB was less stable in water than in DMSO when the copper complex and excess ligands were present. At the molar ratio [RB]/[CuBr_2_]/[Me_6_TREN] = 1/10/30, 15% of RB exists in lactone form, as calculated by the absorption coefficient of RB under these conditions. A separate analysis showed a similar lactonization degree (14%) in the absence of Br–Cu^II^/Me_6_TREN ([RB]/[Me_6_TREN] = 1/20). Interestingly, when only Br–Cu/Me_6_TREN was present ([RB]/[CuBr_2_]/[Me_6_TREN] = 1/10/10), the fraction of the lactone form was 32% (Figure S15). In contrast, RD was quite stable in water in the presence of the Cu catalyst components over a wide range of concentrations (Figure S16). RD-6G showed a similar pattern without any significant change in the absorption because it has an ester group that could not lactonize (Figure S17).

The role and type of excited states involved in the photoreduction mechanism were first examined in DMSO. The excitation of RB generates a singlet excited state, followed by intersystem crossing to a long-lived triplet state. Consequently, this leads to the depletion of the ground-state absorbance of RB, as observed in the kinetic traces after pulsed laser excitation (Figure 4b, red trace). With Br–Cu^II^/Me_6_TREN, only minor ground-state bleaching was observed (Figure 4b, blue trace: ∼4% compared with the absence of Br–Cu^II^/Me_6_TREN). This so-called static quenching occurs when a quencher molecule and a fluorophore form a nonfluorescent complex in the ground state. In this example, the singlet state of RB was oxidatively quenched by Br–Cu^II^/Me_6_TREN efficiently, due to static quenching with recovery of the ground-state RB. This fast electron transfer occurs since the RB and Cu^II^-center are in proximity within the ion pair. This was confirmed by fluorescence quenching analysis.^84^ The fluorescence intensity of RB decreased upon addition of Br–Cu^II^/Me_6_TREN (Figure S18a). The Stern–Volmer plot, where *I*_0_ is the fluorescence in the absence of Br–Cu^II^/Me_6_TREN and *I*_f_ is the fluorescence in the presence of different concentrations of Br–Cu^II^/Me_6_TREN, shows a nonlinear rising slope (Figure S18b, red curve). However, when the fluorescence lifetime of these solutions was measured, no change in the fluorescence lifetime of the RB was observed. Thus, the Stern–Volmer plot of the fluorescence lifetime data gave a line parallel to the *x*-axes (Figure S18b, blue line). These experimental observations indicate the occurrence of static quenching.^84^

Due to its ground-state instability, no investigations of the excited-state properties of RD were performed in DMSO. It was previously reported that RD undergoes electron transfer predominantly from its singlet excited state,^85^ as reflected by a small quantum yield of triplet formation (Φ_isc_ = 0.0024).^76^

RD-6G mainly operates through its singlet excited state (high fluorescence quantum yield, Φ_f_ = 0.90^86^ and negligible i.s.c. yield, Φ_isc_ = 0.002).^78^ When the fluorescence lifetime of RD-6G was monitored in the presence of either Br–Cu^II^/Me_6_TREN or Me_6_TREN, both quenchers decreased the fluorescence lifetimes (Figure 4e). The bimolecular rate constants of singlet state quenching of RD-6G were calculated as *k*_q,ox_^S^ = 5.40 ± 0.09 × 10^9^ and *k*_q,red_^S^ = 2.03 ± 0.06 × 10^9^ M^–1^ s^–1^ for Br–Cu^II^/Me_6_TREN and Me_6_TREN, respectively.

To investigate the influence of the presence of monomer on the quenching rate constants, time-resolved fluorescence quenching experiments of PCs by Me_6_TREN and Br–Cu^II^/Me_6_TREN were also performed in an MA-DMSO mixture (1:1). There were only minor differences, and the overall trend remained the same (Figure S19 and Table 5). The quenching rate constants of RD-6G were *k*_q,ox_^s^ = 8.85 ± 0.31 × 10^9^ and *k*_q,red_^s^ = 2.39 ± 0.03 × 10^9^ M^–1^ s^–1^. In polymerization experiments, the molar ratio of Br–Cu^II^/Me_6_TREN to Me_6_TREN was 1/2, suggesting a slight preference for oxidative quenching (64.9%) for RD-6G.

**Table 5:** Fluorescence Lifetimes (τ_f_), Triplet Quantum Yields (Φ_isc_), and Rate Constants for Singlet Excited-State Reactions (*k*_q_^S^) of PCs with Me_6_TREN and Br–Cu^II^/Me_6_TREN

	RB	RD	RD-6G
τ_f_^DMSO^(ns)	2.25	2.11	3.65
τ_f_^H^_2_^O^(ns)	0.089^88^^,^^a^	1.66	4.19
Φ_isc_	0.40^89^^,^^b^	0.0024^90^	0.002^78^
	0.786^88^^,^^a^		
*k*_q,red_^S^ (10^9^ M^–1^ s^–1^)	0.82 ± 0.04^c^	n.d.^e^	2.03 ± 0.06^c^
	0.48 ± 0.03^d^	1.0 ± 0.1^a^	2.39 ± 0.03^d^
	n.d.^f^		1.53 ± 0.09^a^
*k*_q,ox_^S^ (10^9^ M^–1^ s^–1^)	n.d.^g^	n.d.^e^	5.40 ± 0.09^c^
		13.8 ± 2.6^a^	8.85 ± 0.31^d^
			6.23 ± 0.30^a^
*k*_q,red_^T^ (10^9^ M^–1^ s^–1^)	0.43 ± 0.01^a^		
*k*_q,ox_^T^ (10^9^ M^–1^ s^–1^)	10.4 ± 0.4^a^		

a In water.

b In acetonitrile.

c In DMSO.

d In MA/DMSO (1:1).

e Not determined in DMSO due to lactone formation.

f Not determined in H_2_O due to the very short fluorescence lifetime.

g Not determined due to ion pair formation and static quenching.

The excited properties were also analyzed in aqueous media. Due to its short lifetime (τ_f_ = 0.089 ns in water), the fluorescence properties of RB were not determined. Since RB has high transition yields (Φ_isc_ = 0.786), its triplet state was analyzed. Experiments revealed rapid quenching rates with both Br–Cu^II^/Me_6_TREN (*k*_q,ox_^T^ = 1.04 ± 0.04 × 10^10^ M^–1^ s^–1^) and Me_6_TREN *k*_q,red_^T^ = 4.3 ± 0.1 × 10^8^ M^–1^ s^–1^. Under polymerization conditions ([Br–Cu^II^/Me_6_TREN]/[Me_6_TREN] = 1/2), a strong oxidative quenching mechanism preference was calculated (92.3%) by rate comparison. Similar quenching rate constants in water were observed for RD-6G in the presence of Br–Cu^II^/Me_6_TREN (*k*_q,ox_^S^ = 6.23 ± 0.30 × 10^9^ M^–1^ s^–1^) and Me_6_TREN (*k*_q,red_^S^ = 1.53 ± 0.09 × 10^9^ M^–1^ s^–1^). A stronger preference for oxidative quenching (67.1%) was determined, which is more pronounced than that in DMSO (57.1%). Since RD has a short singlet excited-state lifetime (τ_f_ = 1.66 ns), the efficiency in polymerization experiments should be smaller than that for RD-6G (τ_f_ = 4.19 ns). Bimolecular rate constants of singlet state quenching of RD were determined in water as *k*_q,ox_^S^ = 13.8 ± 2.6 × 10^9^ and *k*_q,red_^S^ = 1.0 ± 0.1 × 10^9^ M^–1^ s^–1^ for Br–Cu^II^/Me_6_TREN and Me_6_TREN, respectively, indicating that oxidative quenching was the major pathway (87.3%). The photophysical properties and bimolecular rate constants of the excited state of the PCs are listed in Table 5. Typical rates of diffusion-controlled reactions in DMSO and H_2_O were estimated as *k*_diff_(DMSO) = 3.3 × 10^9^ and *k*_diff_(H_2_O) = 6.5 × 10^9^ M^–1^ s^–1^, according to eq 4, where *R* is the gas constant (8.314 J·K^–1^·mol^–1^), *T* is the absolute temperature, and η is the viscosity (Pa·s).^87^
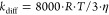
4

These values are close to the rates of electron transfer reactions observed in our systems.

**Scheme 1:**
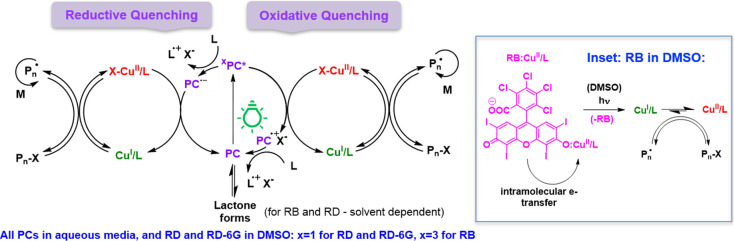
Proposed ATRP Mechanisms with RB, RD, and RD-6G: RB in DMSO Follows an Oxidative Quenching Mechanism by an Intramolecular Electron Transfer through Its Singlet Excited State Due to Ion Pair Formation (Inset) RD and RD-6G in DMSO and all PCs in water follow both oxidative and quenching mechanisms, with oxidative being the major pathway.

The presented results suggest that the contribution of the quenching mechanisms to the overall generation of the activator is solvent-dependent. Based on the spectroscopic data, in DMSO, RB interacts with Br–Cu^II^/Me_6_TREN, forming an ion pair that undergoes static oxidative quenching, predominantly from its singlet state. The concentration of RD diminishes over time due to interactions with the Cu-ATRP catalyst components and lactonization,^83^ preventing its effective regeneration in a photocatalytic cycle under reaction conditions. This leads to higher concentrations of RD needed for PhotoATRP (5 ppm). However, singlet excited states should still be responsible for the redox processes (both oxidative and reductive). RD-6G predominantly participates in redox processes through its singlet excited states following both oxidative and reductive quenching, retaining its overall photostability due to the ester structure and no lactonization. In aqueous media, all PCs prefer oxidative quenching, though with different preference ratios. While RB operates through its triplet state, RD and RD-6G are activated through their singlet states. The overall mechanism of the photoinduced electron transfer processes is illustrated in Scheme 1.

## Conclusions

In conclusion, three commercially available xanthene dyes, rose bengal (RB), rhodamine B (RD), and rhodamine 6G (RD-6G), were tested at variable concentrations as PCs in dual-catalytic PhotoATRP. 50 ppm Br–Cu^II^/Me_6_TREN catalysts provided good control over molecular weights and dispersities. Photoexcited dyes regenerated Cu^I^/L activators and controlled the polymerization rate, offering excellent temporal control. RB and RD-6G enabled regenerative ATRP even at subppm concentrations ([PC] ≥ 10 ppb), whereas RD required a minimum of 5 ppm due to its significant lactonization.

Based on the presented mechanistic studies, the Cu^I^/L photoregeneration depends on the structure of xanthene dyes. In DMSO, RB forms an ion pair with Cu^II^/Me_6_TREN that undergoes oxidative static quenching, predominantly from a singlet excited state. RD-6G cannot lactonize and follows a mixed oxidative and reductive quenching mechanism from its singlet excited state. RD follows a mixed quenching mechanism from its excited singlet state and requires higher concentrations than the other tested PCs due to significant lactonization. In water, all PCs follow a mixed quenching mechanism with the dominant oxidative pathway. While RB undergoes triplet state quenching, RD and RD-6G are reactive in their excited singlet states.

Polymers with well-controlled molecular weights, narrow molecular weight distributions, and high chain-end fidelity, as evidenced by the efficient chain extension, were prepared using 50 ppm of Cu catalysts and ppb amounts of xanthene cocatalysts.

## Experimental Section

### Materials

Unless otherwise noted, all chemicals were purchased from commercial sources and used as received. Rose bengal (RB, 95%), rhodamine B (RD, ≥95%), rhodamine 6G (RD-6G, 99%), copper(II) bromide (CuBr_2_, 99.99%), ethyl α-bromoisobutyrate (EBiB, 99%), 2-hydroxyethyl α-bromoisobutyrate (HO-EBiB, 95%), methyl acrylate (MA, 99%), ethyl acrylate (EA, ≥99.5%), methoxyethyl acrylate (MEA, 98%), and oligo(ethylene glycol) methyl ether methacrylate (average *M* = 500, OEOMA_500_) were purchased from Sigma-Aldrich. All monomers were passed through a column of basic alumina to remove the inhibitor prior to the use. Tris(2-pyridylmethyl)amine (TPMA, 99%) and tris[2-(dimethylamino)ethyl]amine (Me_6_TREN, 99%) were purchased from Ambeed. Water (distilled), dimethylformamide (DMF, HPLC grade), tetrahydrofuran (THF, HPLC grade), acetonitrile (ACN, HPLC grade), and dimethyl sulfoxide (DMSO, HPLC grade) were purchased from Fisher Chemical. D_2_O and DMSO-*d*_6_ were purchased from Cambridge Isotope Laboratories, Inc.

### Instrumentation

#### Photoreactor for PhotoATRP

Polymerization was conducted in an EvoluChemTM PhotoRedOx Box purchased from Hepatochem with varying LEDs. LEDs with green light (525 nm, 25 mW·cm^–2^) and red light (640 nm, 25 mW·cm^–2^) were purchased from Kessil.

#### ^1^H Nuclear Magnetic Resonance (^1^H NMR)

^1^H NMR spectra were recorded on a Bruker Avance III 500 MHz spectrometer with D_2_O or DMSO-*d*_6_ used as the solvent.

#### Size Exclusion Chromatography (SEC)

The molecular weights (*M*_n_) and dispersities (*Đ*) were measured relative to poly(methyl methacrylate) (PMMA) standards by gel permeation chromatography (GPC) conducted with an Agilent GPC instrument using THF as the eluent. The GPC was equipped with an RI detector and PSS columns (Styrogel 105, 103, 102 Å) at 35 °C and a flow rate of 1 mL/min.

#### Spectroscopy

UV–vis spectra were recorded on an Agilent 8453 spectrometer.

Fluorescence lifetime measurements were performed by time-correlated single photon counting using an FLS1000 spectrometer (Edinburgh Analytical Instruments) in conjunction with a pulsed LED (496 nm from PicoQuant).

Laser flash photolysis experiments employed the pulses from a Spectra-Physics GCR-150-30 Nd:YAG laser (532 nm, 7 ns pulse width) and a computer-controlled system that was described previously.^91^

#### Electrochemistry

Cyclic voltammetry was performed using a CH Instruments Electrochemical Analyzer 600C potentiostat with a three-electrode system consisting of a silver wire pseudoreference electrode, a platinum coil counter electrode, and a glassy carbon working electrode. Experiments were performed at 1 mM analyte concentration with 0.1 M tetrabutylammonium hexafluorophosphate as the supporting electrolyte in acetonitrile after purging the solution with argon until no traces of oxygen signals were visible in the CV. Voltammograms were collected using a scan rate of 0.1 V/s, and redox potentials were referenced to a ferrocene internal standard (*E*^0^ (Fc^+^/Fc) = 0.40 V vs SCE in ACN). Experiments revealed an irreversible oxidation pattern for Me_6_TREN. The oxidation peak potential of Me_6_TREN was 0.51 V vs SCE in ACN.

#### General Procedure for RB, RD, RD-6G/Cu-Catalyzed Photo-ATRP of MA under Green Light Irradiation

All the polymerizations were performed by employing the same procedure as described below for the case of RD-6G.

The reaction mixtures were prepared in a 5 mL volumetric flask. MA (2.5 mL, 27.8 mmol), CuBr_2_ (1.4 μmol, added as 20.2 μL from a stock solution of 68.7 mM in DMSO), Me_6_TREN (4.17 μmol, added as 11 μL from a stock solution of 372 mM in DMSO), RD-6G (0.14 μmol, added as 19 μL from a stock solution of 7.4 mM in DMSO), and EBiB (54 mg, 0.278 mmol) were added. DMSO was added to fill up the volume to 5 mL. The reaction mixtures were transferred to a 2-dram pressure release vial charged with a stir bar under a N_2_ atmosphere. The polymerizations started upon turning on the green LEDs (525 nm, 25 mW·cm^–2^). The mixtures were stirred at 300 rpm for 90 min. Samples were collected with a syringe and analyzed using ^1^H NMR and GPC techniques. Aliquots were taken at different times under a blanket of N_2_ to analyze the reaction’s kinetics.

#### Procedure for RB/Cu-Catalyzed Photo-ATRP of OEOMA_500_ in Aqueous Media

Stock solutions of the emulsified OEOMA_500_ (600 mM in H_2_O), PCs (1.88 mM in H_2_O), HO-EBiB (75 mM in DMSO), CuBr_2_ (11.2 mM in H_2_O), and TPMA (67.49 mM in DMSO) were prepared. A typical ATRP solution (250 μL) using RB was then prepared by mixing OEOMA_500_ stock (125 μL), RB stock (5 μL), CuBr_2_ stock (10 μL), TPMA stock (5 μL), HO-EBiB stock (5 μL), DMSO (15 μL), H_2_O (60 μL), and 10× PBS solution (25 μL). The final concentrations were OEOMA_500_ (300 mM), RB (37.5 μM), CuBr_2_ (0.45 mM), TPMA (1.35 mM), and HO-EBiB (1.5 mM). The ATRP solution was then transferred to a 96-well plate, which was mounted on a Lumidox Gen II 96-point LED array. The ATRP mixtures were irradiated under green LEDs (525 nm, 25 mW·cm^–2^) for 60 min. Samples were withdrawn for ^1^H NMR and GPC characterization.

#### Chain Extension

Chain extensions were performed using the same procedure as the polymerizations described above, employing PMA-Br synthesized as a macroinitiator under otherwise the same conditions. All polymers were precipitated in a methanol/water mixture and dried under a vacuum before use.

#### Varying Degrees of Polymerization

The targeted degree of polymerization (DP_target_ = 200, 400, 800, and 1600) was varied by adjusting the EBiB concentration with respect to the monomer concentration. The polymerizations were conducted by the same procedure as described above.

## Supporting Information Available

Tables summarizing the polymerization results performed with PMDETA as an excess ligand and with Me_6_TREN for varying degrees of polymerization (DP), table showing the results for other acrylic monomers, chain extensions, and kinetics at 100 ppb PCs, GPC curves of polymers with different targeted DPs, UV–vis spectra of the PCs at higher concentrations, UV–vis spectra of RB in the presence/absence of Br–Cu/Me_6_TREN in DMSO and water, and UV–vis spectra of RD and RD-6G in the presence/absence of Br–Cu/Me_6_TREN in DMSO (PDF)
