# Supplementary material for: ATRP with ppb Concentrations of Photocatalysts
Source: J Am Chem Soc. 2024 Oct 10;146(42):28994–9005. doi: 10.1021/jacs.4c09927 (PMC11503771; doi:10.1021/jacs.4c09927)
Supplement: Supplementary file 1 — ja4c09927_si_001.pdf [file ja4c09927_si_001.pdf]

Supporting Information

ATRP with ppb Concentrations of Photocatalysts

Halil Ibrahim Coskun,[a] Ferdinando De Luca Bossa,[a] Xiaolei Hu,[a] Steffen Jockusch,[b] Julian Sobieski,[a] Gorkem Yilmaz, [a*] Krzysztof Matyjaszewski [a*]

[a] Department of Chemistry, Carnegie Mellon University, 4400 Fifth Avenue, Pittsburgh, Pennsylvania 15213, United States

[b] Department of Chemistry and Center for Photochemical Sciences, Bowling Green State University, Bowling Green, Ohio 43403, United States

**Table of Content**	**Page**
Figure S1. a) Kinetics of photoATRP of MA RD-6G, b) M_n vs Conv. (%), using 100 ppb	3
Figure S2. GPC traces of the polymers given in Table 1 in the main text	4
Table S1. Photoinduced ATRP of MA using EBiB and $CuBr_2$/PMDETA under different conditions	5
Figure S3. GPC traces of the polymers obtained by using PMDETA as the excess ligand	5
Figure S4. The effect of [PC], [$CuBr_2$], and [free Me_6TREN] on the polymerization rate of MA	6
Table S2. Photoinduced ATRP of acrylic monomers using EBiB and $CuBr_2$/Me_6TREN under different conditions[a]	7
Figure S5. GPC traces of the polymers obtained according to the conditions given in Table S2	8
Table S3. Polymerizations at varying targeted DPs	9
Figure S6. GPC traces of PMAs, prepared by targeting various degrees of polymerizations using 5 ppm PCs	10
Figure S7. GPC traces of PMAs, prepared by targeting various degrees of polymerizations using 100 ppb RB and RD-6G	10
Figure S8. UV spectra of the PCs at different concentrations	11
Figure S9. a) Temporal control with 100 ppb RD-6G b) b) chain end fidelity using 100 ppb RD-6G and RB	11
Figure S10. UV-Vis Spectra of the PCs in DMSO and water	12

Figure S11. UV-Vis spectra and visual appearance of RB in the presence/absence of $CuBr_2$ and Me_6TREN in DMSO 12
Figure S12. UV spectra of RB at $1x10^{-5}$ M concentration in the presence/absence of $CuBr_2$/Me_6TREN (1:1) Complex in DMSO and DI Water. 13
Figure S13. UV-Vis spectra and visual appearance of RD in the presence/absence of $CuBr_2$ and Me_6TREN with different stoichiometry in DMSO 13
Figure S14. UV-Vis spectra and visual appearance of RD-6G in the presence/absence of $CuBr_2$ and Me_6TREN in DMSO 14
Figure S15. UV-Vis spectra and visual appearance of RB in the presence/absence of $CuBr_2$ and Me_6TREN with different stoichiometry in water 14
Figure S16. UV-Vis spectra and visual appearance of RD in the presence/absence of $CuBr_2$ and Me_6TREN with different stoichiometry in water 15
Figure S17. UV-Vis spectra and visual appearance of RD-6G in the presence/absence of $CuBr_2$ and Me_6TREN with different stoichiometry in water 15
Figure S18: a) Fluorescence spectra of RB (6.5 μM) in DMSO in the absence and presence of varying concentrations of $CuBr_2$/Me_6TREN (1:1) (λ_{ex} = 520 nm). b) Stern-Volmer plots of steady-state fluorescence intensity (I_0 is the fluorescence in the absence of quencher and I_f is the fluorescence intensity in the presence of quencher) using data shown in a) (red) and Stern-Volmer plot of fluorescence lifetime measurements (λ_{ex} = 496 nm, pulsed LED; λ_{em} = 585 nm) determined by time-correlated single photon counting where τ_0 is the fluorescence in the absence of quencher and τ_f is the fluorescence intensity in the presence of quencher (blue). 16
Figure S19 a) Determination of the bimolecular rate constants k_q^s of quenching of RB singlet excited states by Me_6TREN in MA/DMSO (1:1) using fluorescence lifetime measurements (λ_{ex} = 496 nm, pulsed LED). Inverse fluorescence lifetime of RB (λ_{em} = 585 nm) determined by time-correlated single photon counting vs. varying concentration of quencher, b) Determination of the bimolecular rate constants k_q^s of quenching of RD-6G singlet excited states by Me_6TREN (red) and $CuBr_2$/Me_6TREN (1:1) (blue) in MA/DMSO (1:1) fluorescence lifetime measurements (λ_{ex} = 496 nm, pulsed LED). Inverse fluorescence lifetime of RD-6G (λ_{em} = 568 nm) determined by time-correlated single photon counting vs. varying concentration of quencher. 17

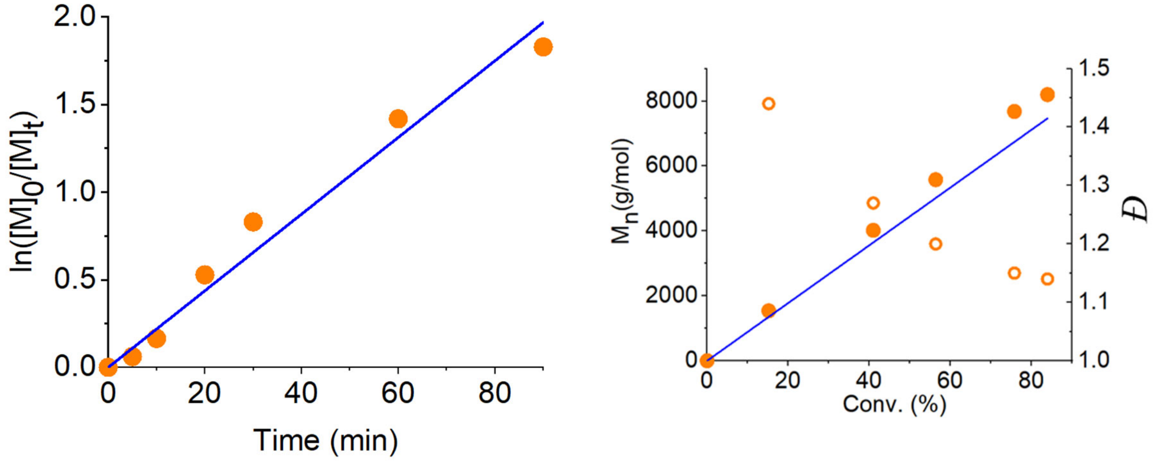

Figure S1. a) Kinetics of MA polymerization using RD-6G, b) M_n vs Conv. (%) plot (Conditions: [MA]/[EBiB]/[$CuBr_2$]/[Me_6TREN]/[RD-6G] = 100/1/0.005/0.015/0.00001, 100 ppb of PC V_{MA}/V_{DMSO}: 1/1, λ ~ 525 nm, under N_2 atmosphere)

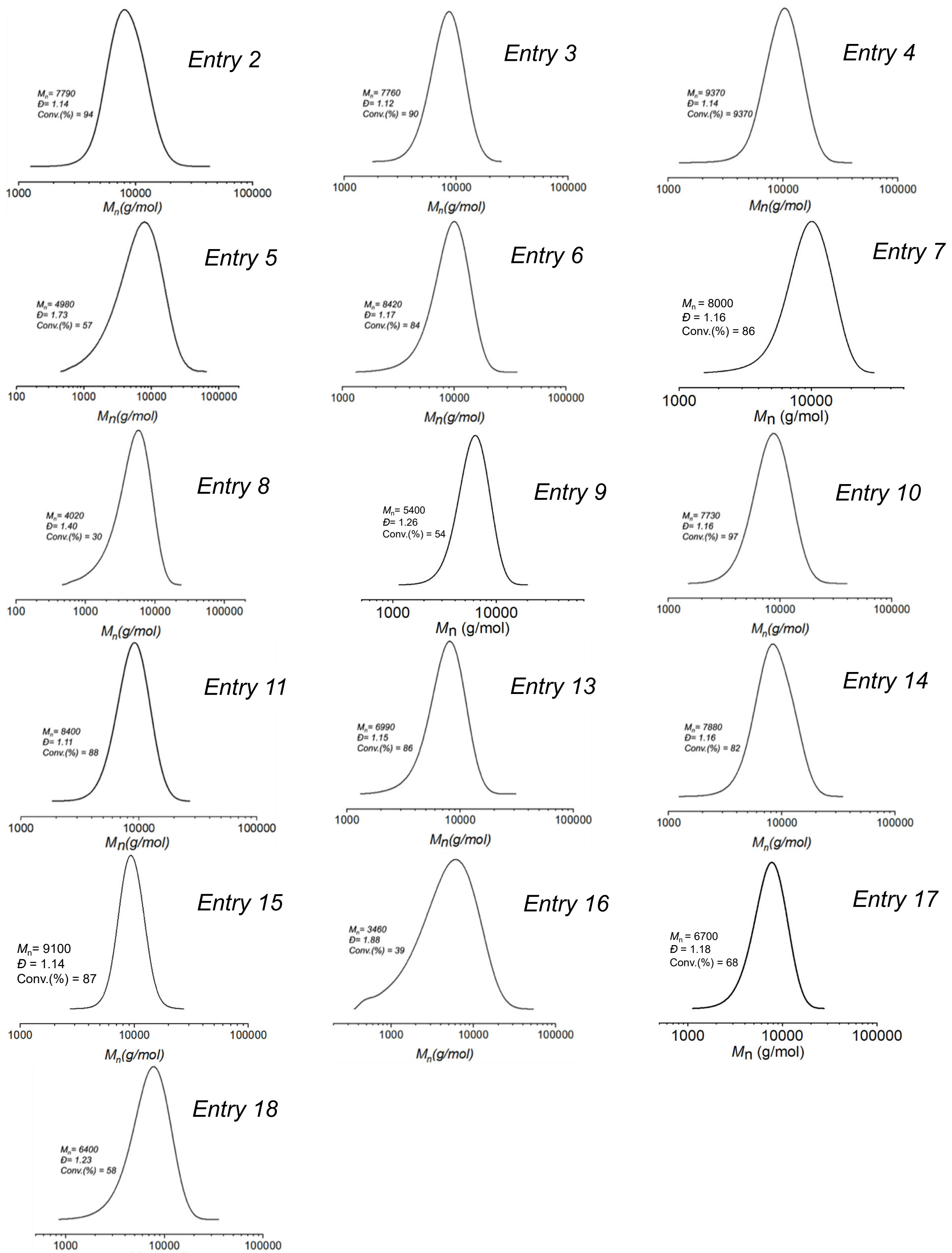

Figure S2. GPC traces of the polymers from Table 1

Table S1. Photoinduced ATRP of MA using EBiB and $CuBr_2$/PMDETA under different conditions[a]

Entry	PC	$[CuBr_2]$/[PMDETA]/[PC]	[PC] (ppm)	Conv. (%)[b]	$M_{n,theo}$ ($g \cdot mol^{-1}$)[c]	$M_{n,GPC}$ ($g \cdot mol^{-1}$)[d]	$Đ$[d]	I[e]
1	RB	0.02/0.2/0.001	10	97	8540	14550	1.13	0.59
2		0.04/0.2/0.004	40	86	7590	9080	1.09	0.84
3		0.04/0.1/0.004	40	73	6470	6730	1.09	0.96
4	RD	0.02/0.2/0.001	10	98	8620	5280	1.19	1.63
5		0.1/0.1/0.0005	5	97	8540	6140	1.20	1.39

[a] [MA]/[EBiB]/$[CuBr_2]$/[PMDETA]/[PC]: 100/1/x/y/z, V_{MA}/V_{DMSO}: 1/1, (λ ~ 525 nm, intensity: 25 $mW \cdot cm^{-2}$)

[b] Determined by ^{1}H NMR [c] $M_{n,theo}$= Conv(%) × 85+ 195 [d] Determined by GPC in THF using polymethyl methacrylate standards

[e] Initiation efficiency (I) = $M_{n,theo}$/ $M_{n,GPC}$

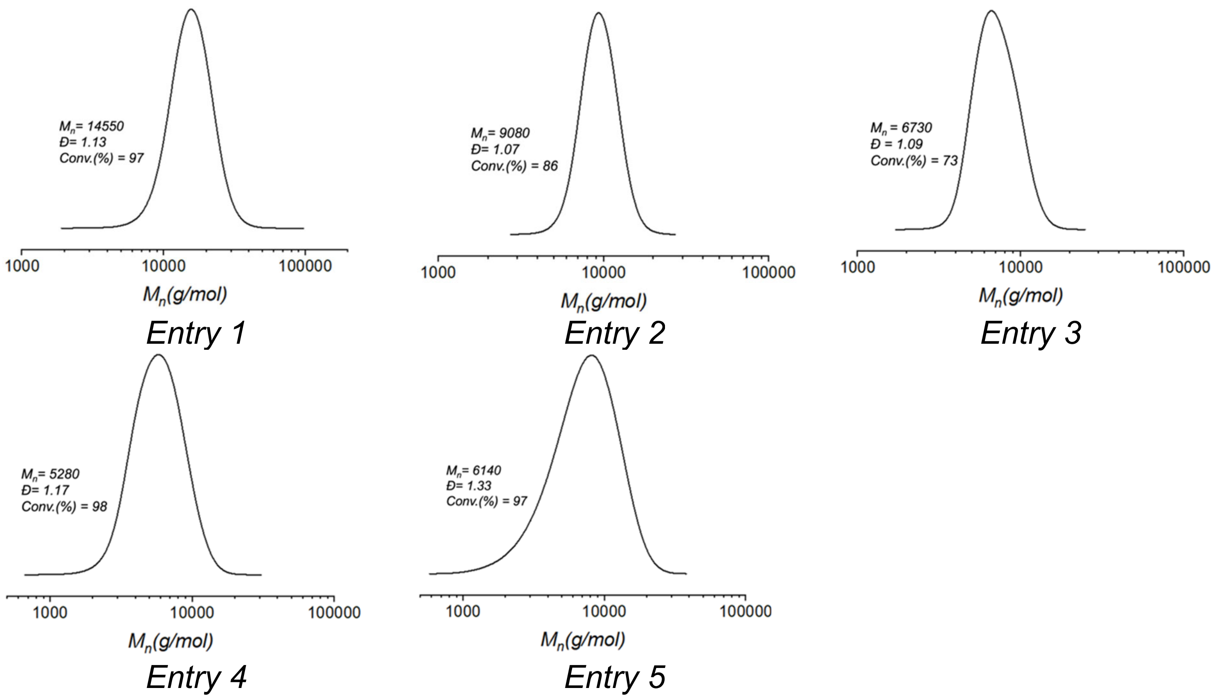

Figure S3. GPC traces of the polymers obtained by using PMDETA as the excess ligand

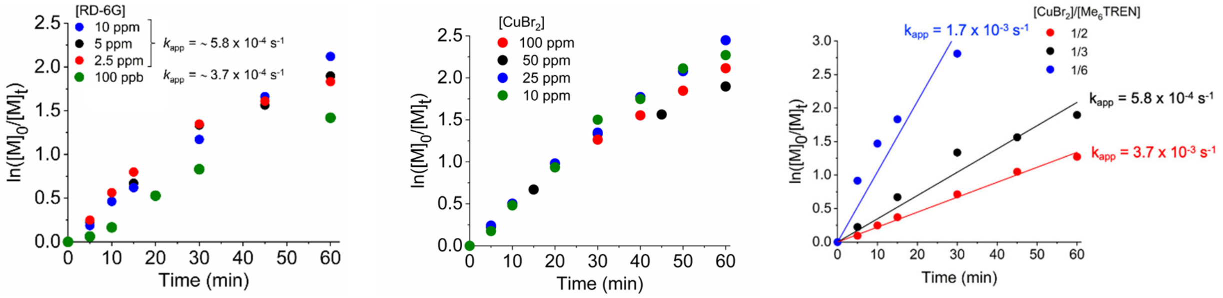

Figure S4. The effect of [RD-6G] ([MA]/[EBiB]/[$CuBr_2$]/[Me_6TREN]/[PC]: 100/1/0.005/0.015/x, [$CuBr_2$] = 50 ppm), [$CuBr_2$] ([MA]/[EBiB]/[$CuBr_2$]/[Me_6TREN]/[PC]: 100/1/x/y/0.0005, [RD-6G] = 5 ppm) and [free Me_6TREN] on the polymerization rate: [MA]/[EBiB]/[$CuBr_2$]/[Me_6TREN]/[PC]: 100/1/0.005/x/0.0005, [RD-6G] = 5 ppm), For all reactions: V_M/V_{DMSO} = 1/1, under N_2 (λ ~ 525 nm, intensity: 25 mW·cm^{-2}).

Table S2. Photoinduced ATRP of acrylic monomers using EBiB and $CuBr_2$/Me_6TREN under different conditions[a]

Entry	PC	[M][b]	[PC] (ppb)	Time (h)	Conv. (%)[c]	$M_{n,theo}$ (g·mol^{-1})[d]	$M_{n,GPC}$ (g·mol^{-1})[e]	$Đ$[e]	I[f]
**1**	RB	MA*	100	10	89	7850	9000	1.14	0.87
**2**		EA	100	10	61	6300	11000	1.14	0.57
**3**		MEA	100	10	81	10900	13200	1.17	0.83
**4**	RD	MA*	5000	6	88	7760	8400	1.11	0.92
**5**		EA	5000	6	79	8100	10100	1.14	0.80
**6**		MEA	5000	6	90	12100	12700	1.15	0.95
**7**	RD-6G	MA	100	1.5	87	7700	9100	1.14	0.85
**8**		EA	100	1.5	75	7700	9000	1.16	0.86
**9**		MEA	100	1.5	85	11400	11600	1.18	0.98

[a] [M]/[EBiB]/[$CuBr_2$]/[Me_6TREN]/[PC]: 100/1/0.005/0.015/x, [$CuBr_2$] = 50 ppm V_M/V_{DMSO} = 1/1, under N_2 (λ ~ 525 nm, intensity: 25 mW·cm^{-2})

[b] Determined by ^{1}H NMR [c] $M_{n,theo}$= Conv(%) × M_M+ 195 [d] Determined by GPC in THF using polymethyl methacrylate standards, [e] Initiation efficiency (I) = $M_{n,theo}/M_{n,GPC}$.

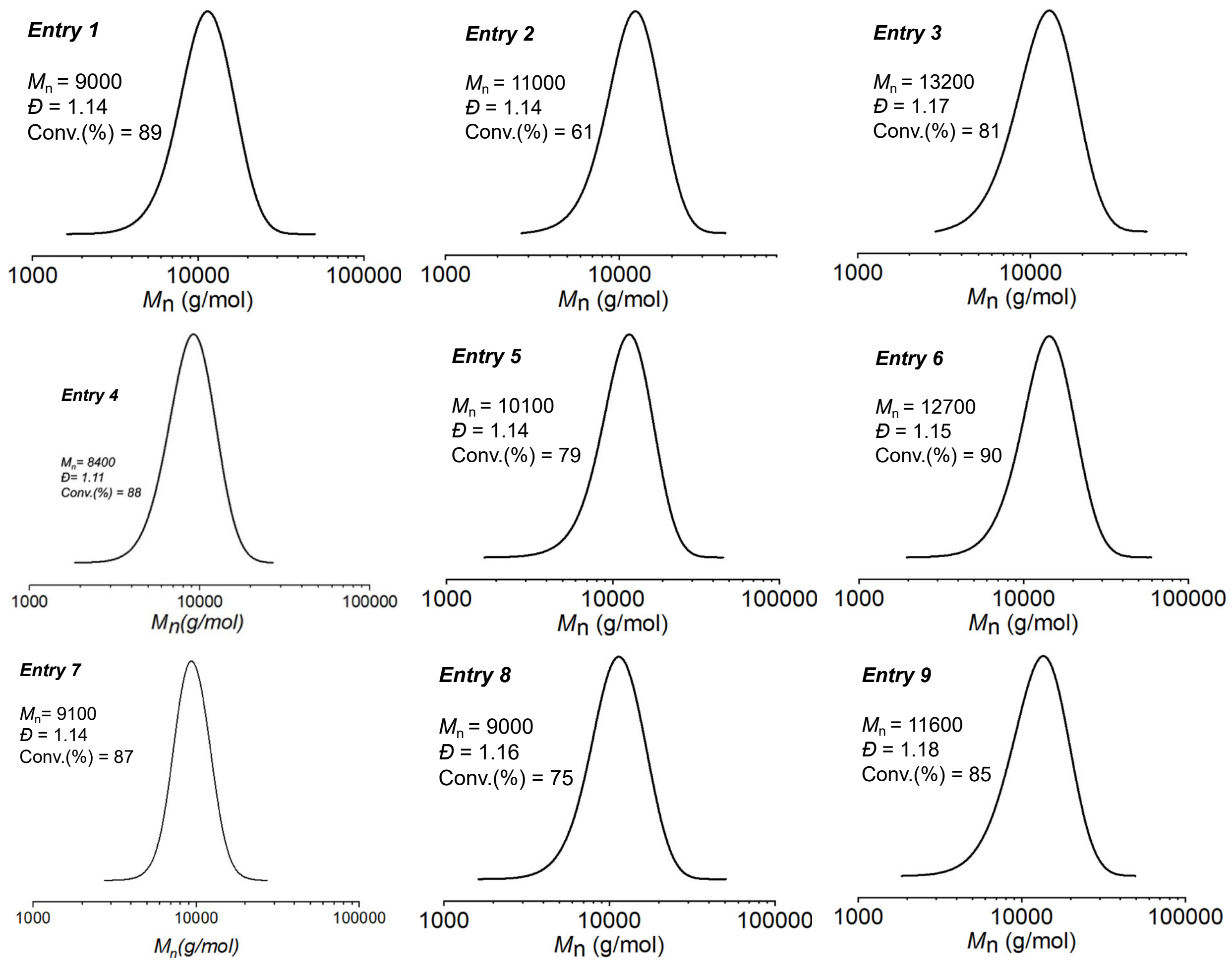

Figure S5. GPC traces of the polymers obtained according to the conditions given in Table S2

Table S3. Photoinduced ATRP of MA using EBiB and $CuBr_2$/Me_6TREN with varying degree of polymerization[a]

PC	[PC] (ppb)	DP	Time (h)	Conv. (%)[b]	$M_{n,theo}$ ($g\cdot mol^{-1}$)[c]	$M_{n,GPC}$ ($g\cdot mol^{-1}$)[d]	$Đ$[d]	I[e]
RB	100	200	10	94	16200	18200	1.08	0.89
		400	10	97	33400	31900	1.06	1.05
		800	10	90	61900	62200	1.04	0.96
		1600	10	77	106000	108600	1.03	0.98
	5000	200	3	82	14300	14800	1.08	0.97
		400	3	62	21520	22430	1.07	0.96
		800	3	74	51100	52670	1.04	0.97
		1600	3	57	78630	77140	1.04	1.02
RD	5000	200	8	83	14470	14460	1.08	~1.00
		400	8	95	32890	30270	1.06	1.09
		800	21	96	66240	64260	1.07	1.03
RD-6G	100	200	3	83	14300	16500	1.08	0.87
		400	3	88	30300	27500	1.06	1.10
		800	3	81	55700	60000	1.04	0.93
		1600	3	67	92200	106500	1.05	0.87
	5000	200	1.5	96	16700	18360	1.08	0.91
		400	1.5	97	33560	33510	1.06	~1.00
		800	1.5	91	62800	71690	1.06	0.88
		1600	1.5	90	124000	142240	1.08	0.87

[a] [MA]/[EBiB]/[$CuBr_2$]/[Me_6TREN]/[PC] = 100/x/0.005/0.015/y, [$CuBr_2$] = 50 ppm, V_{MA}/V_{DMSO} = 1/1, (λ ~ 525 nm, intensity: 25 $mW\cdot cm^{-2}$)

[b] Determined by ^1H NMR [c] $M_{n,theo}$= Conv(%) × 85+ 195 [d] Determined by GPC in THF using polymethyl methacrylate standards

[e] Initiation efficiency (I) = $M_{n,theo}$/ $M_{n,GPC}$

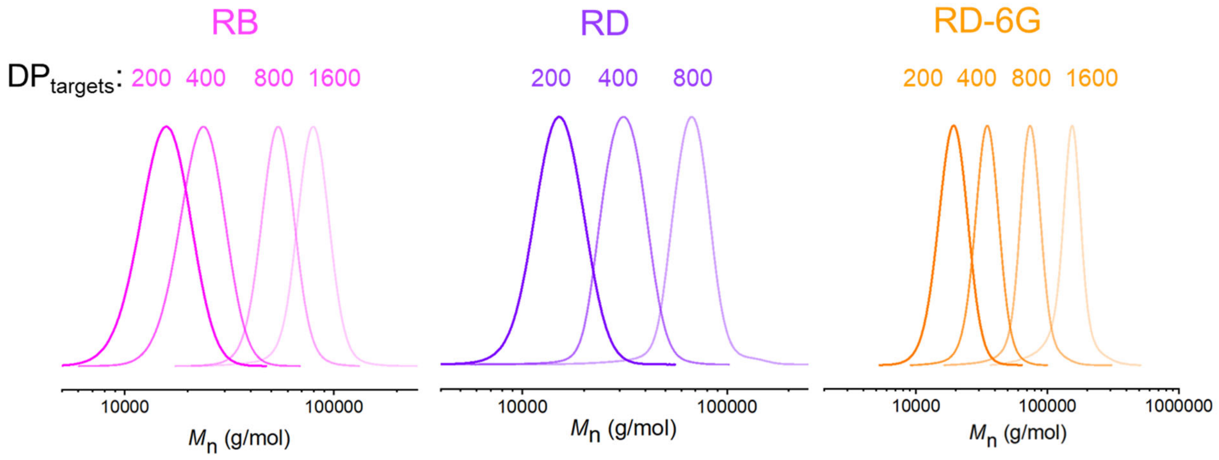

Figure S6. GPC traces of PMAs, prepared by targeting various degrees of polymerizations using 5 ppm PCs

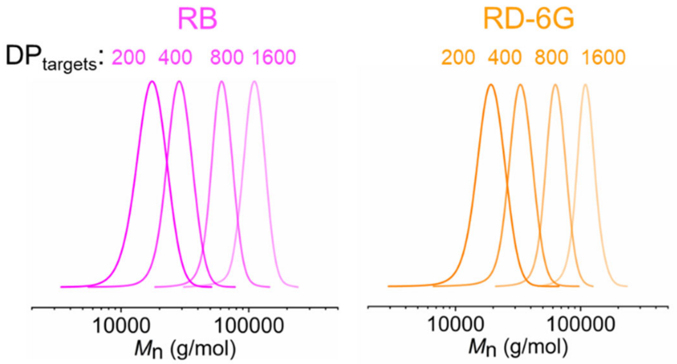

Figure S7. GPC traces of PMAs, prepared by targeting various degrees of polymerizations using 100 ppb RB and RD-6G

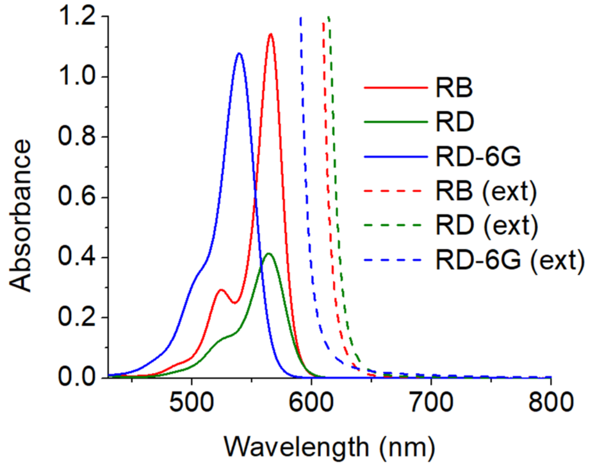

Figure S8. UV spectra of the photocatalysts at different concentrations in DMSO ([RB]:10^{-5}M, [RD]: 3×10^{-5}M, [RD-6G]: 1.5×10^{-5}M. For tail absorptions at extended region (dots), $[PC]_{ext} = 400 \times [PC]$)

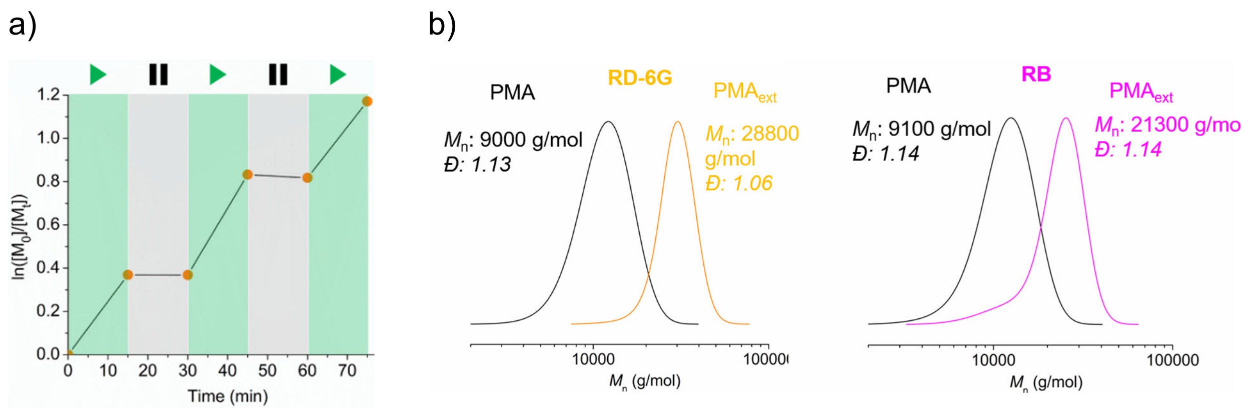

Figure S9. a) Temporal control with 100 ppb RD-6G ([MA]/[EBiB]/[$CuBr_2$]/[Me_6TREN]/[RD-6G] = 100/1/0.005/0.015/0.00001, [$CuBr_2$] = 50 ppm, and b) chain end fidelity using 100 ppb RD-6G and RB
(For [PMA] : [MA]/[EBiB]/[$CuBr_2$]/[Me_6TREN]/[PC] = 100/1/0.005/0.015/0.00001, [$CuBr_2$] = 50 ppm, For $[PMA]_{ext}$: [MA]/[PMA]/[$CuBr_2$]/[Me_6TREN]/[PC] = 300/1/0.015/0.045/0.00003, [$CuBr_2$] = 50 ppm).

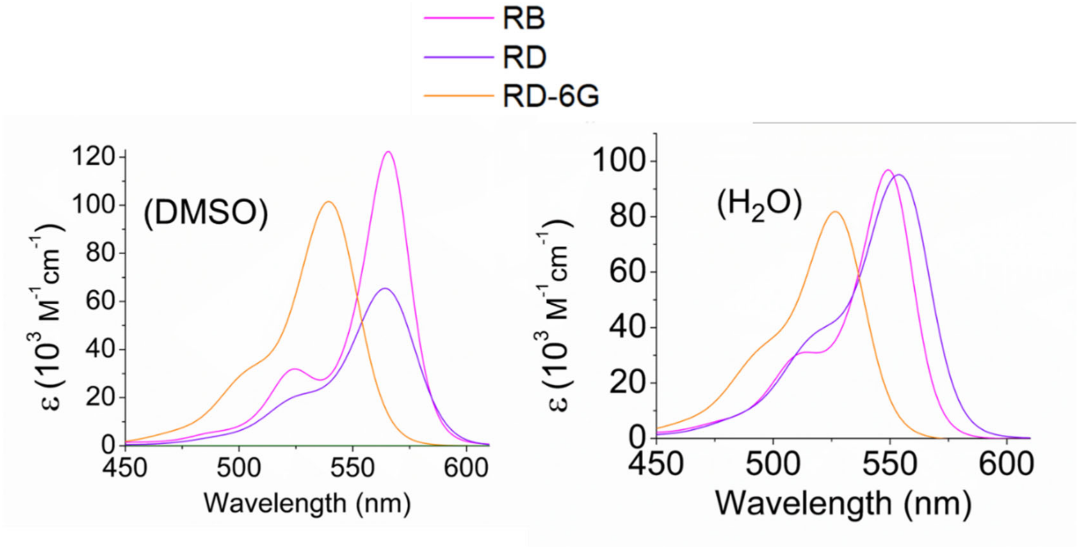

Figure S10. UV-Vis spectra of the PCs in DMSO and water. Absorption coefficients were calculated using the formula, A = ε·C·l (A: absorbance, ε = Absorption coefficient, l = length of the UV cell (1 cm)): $\varepsilon_{RB,(DMSO)}$ = 122400, $\varepsilon_{RD,(DMSO)}$ = 65400, $\varepsilon_{RD\text{-}6G,(DMSO)}$ = 101500, $\varepsilon_{RB,(H2O)}$ = 96900, $\varepsilon_{RD,(H2O)}$ = 95300, $\varepsilon_{RD\text{-}6G,(H2O)}$ = 81800

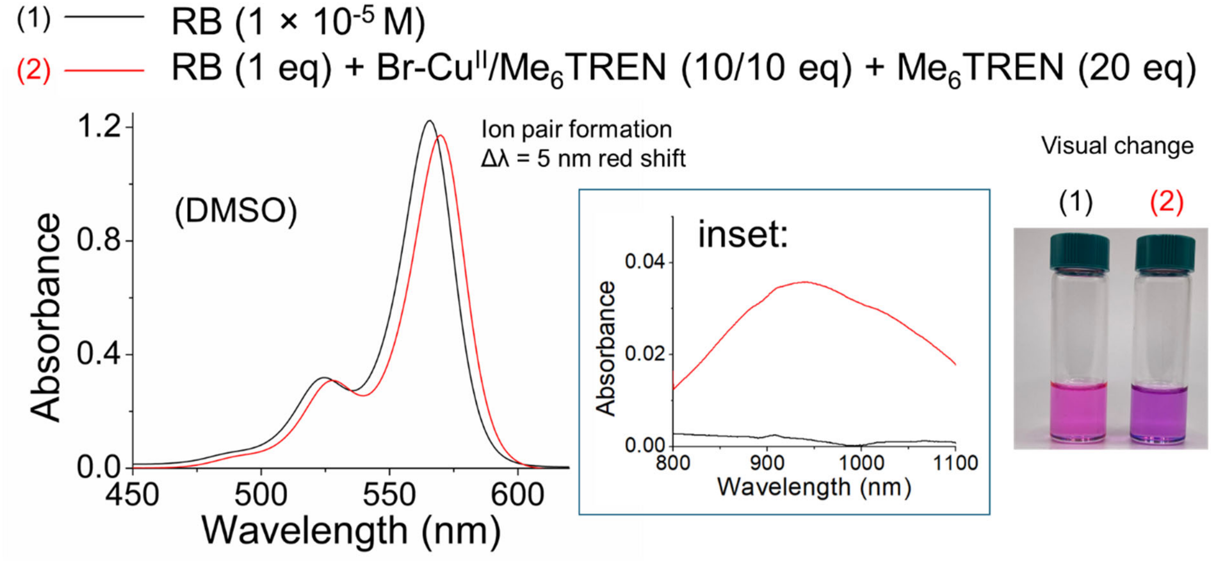

Figure S11. UV-Vis spectra and visual appearance of RB in the presence/absence of $CuBr_2$ and Me_6TREN in DMSO

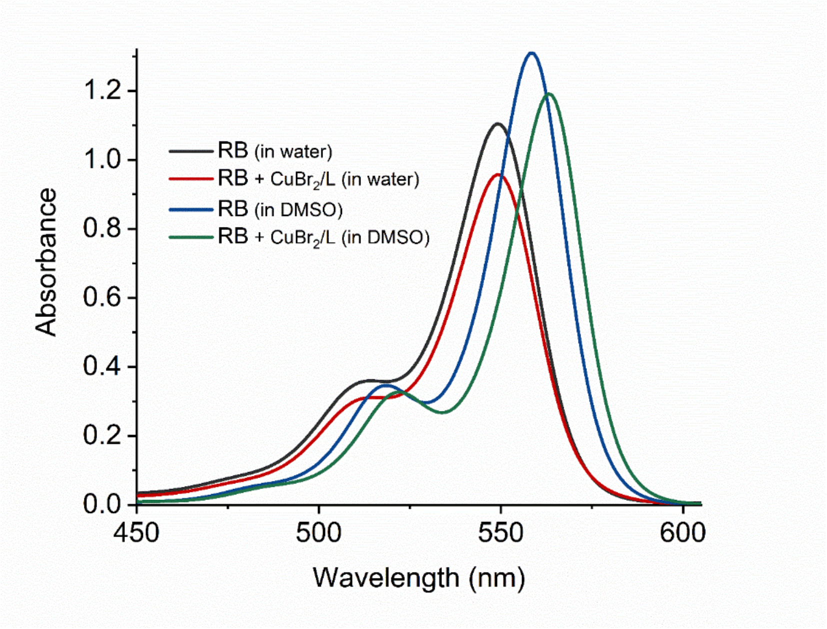

Figure S12. UV spectra of RB at 1x10^{-5} M concentration in the presence/absence of $CuBr_2$/Me_6TREN (1:1) Complex in DMSO and DI Water.

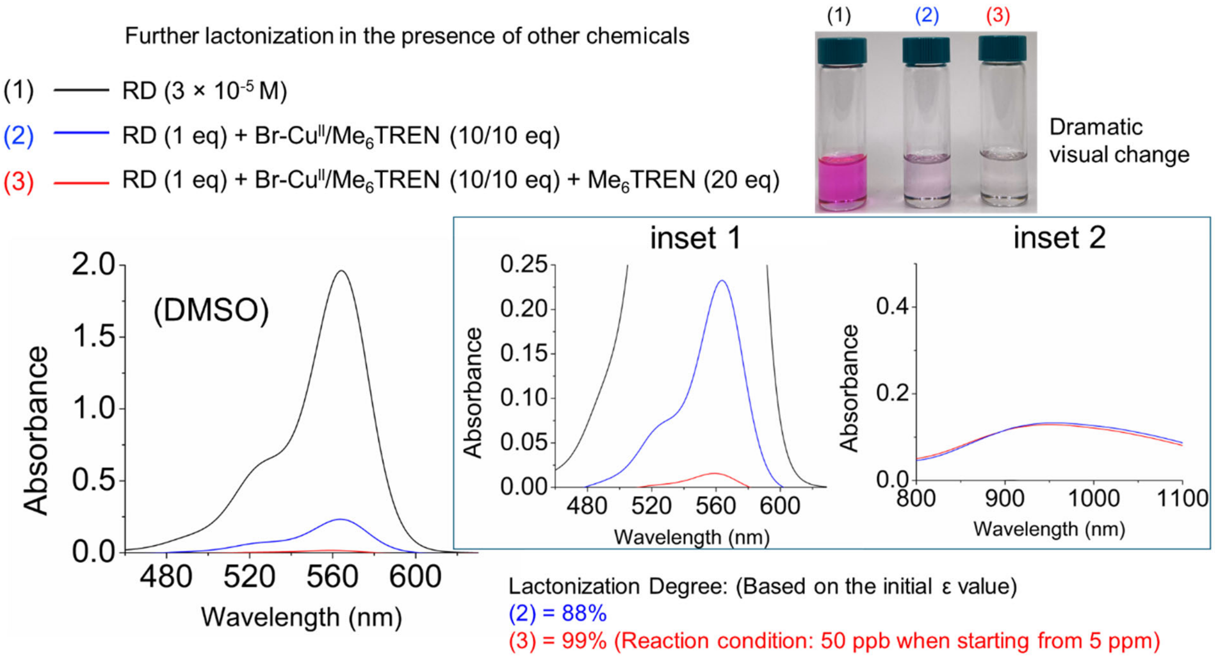

Figure S13. UV-Vis spectra and visual appearance of RD in the presence/absence of $CuBr_2$ and Me_6TREN at different stoichiometry in DMSO.

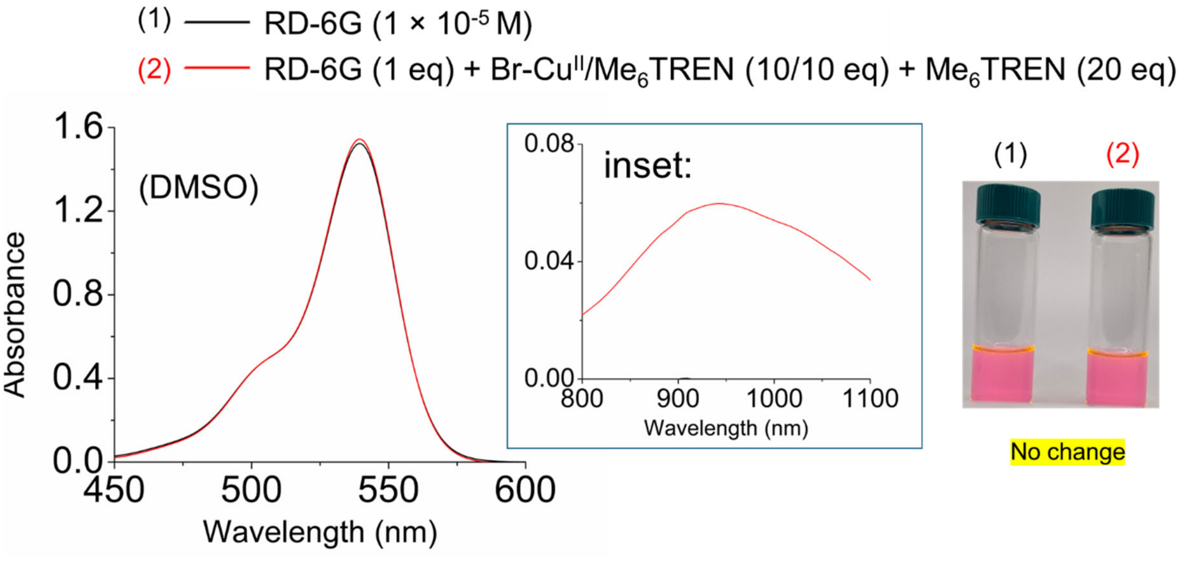

Figure S14. UV-Vis spectra and visual appearance of RD-6G in the presence/absence of $CuBr_2$ and Me_6TREN in DMSO

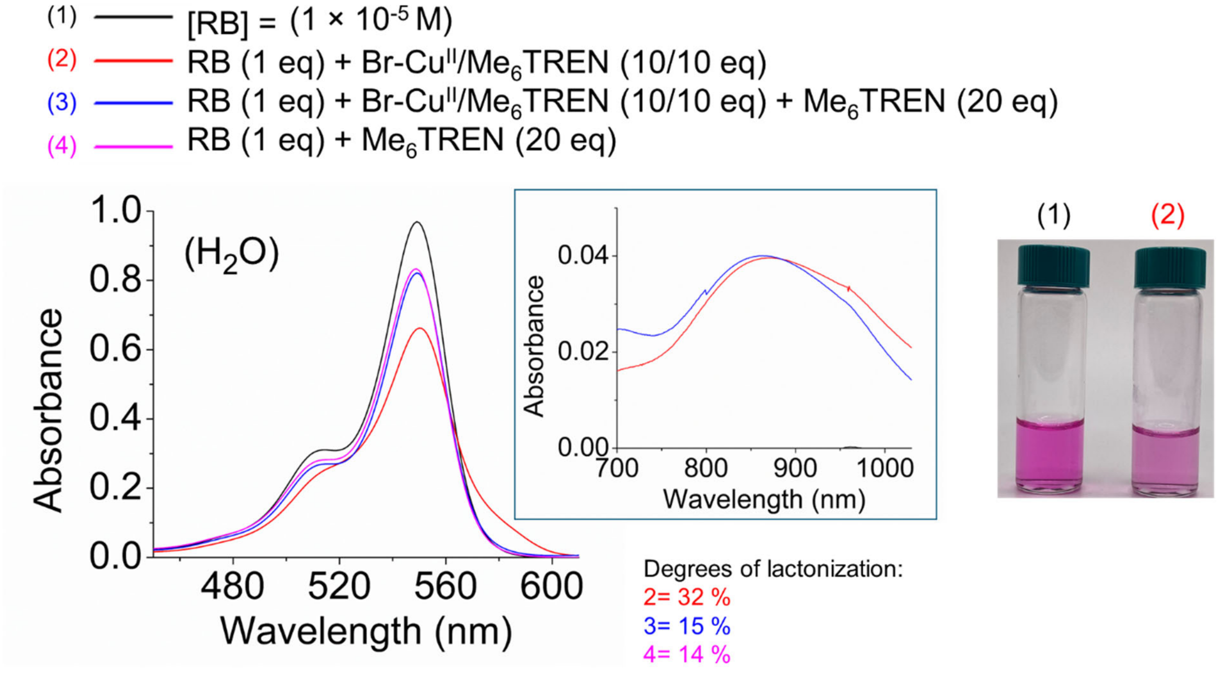

Figure 15. UV-Vis spectra and visual appearance of RB in the presence/absence of $CuBr_2$ and Me_6TREN at different stoichiometry in water

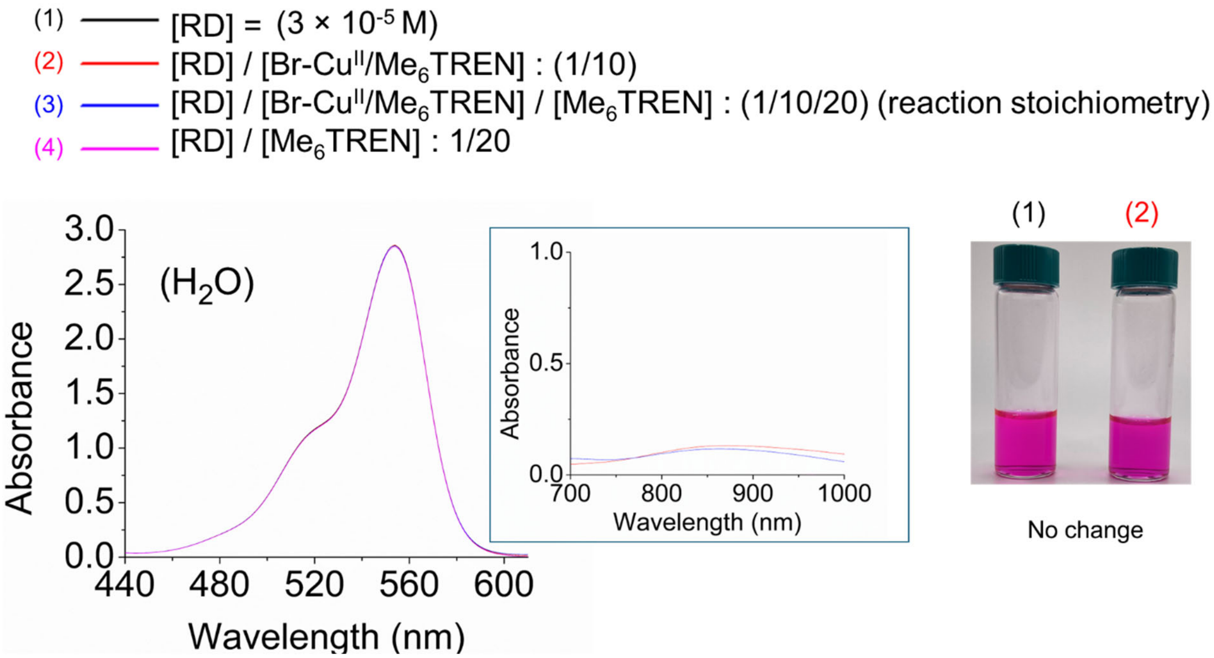

Figure S16. UV-Vis spectra and visual appearance of RD in the presence/absence of $CuBr_2$ and Me_6TREN at different stoichiometry in water.

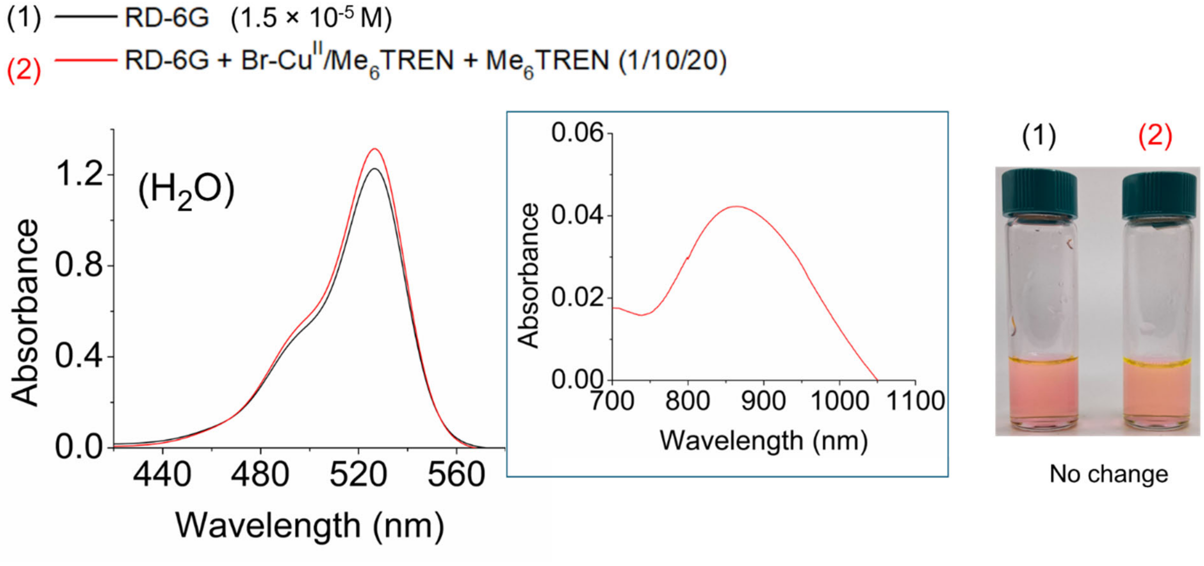

Figure S17. UV-Vis spectra and visual appearance of RD-6G in the presence/absence of $CuBr_2$ and Me_6TREN at different stoichiometry in water.

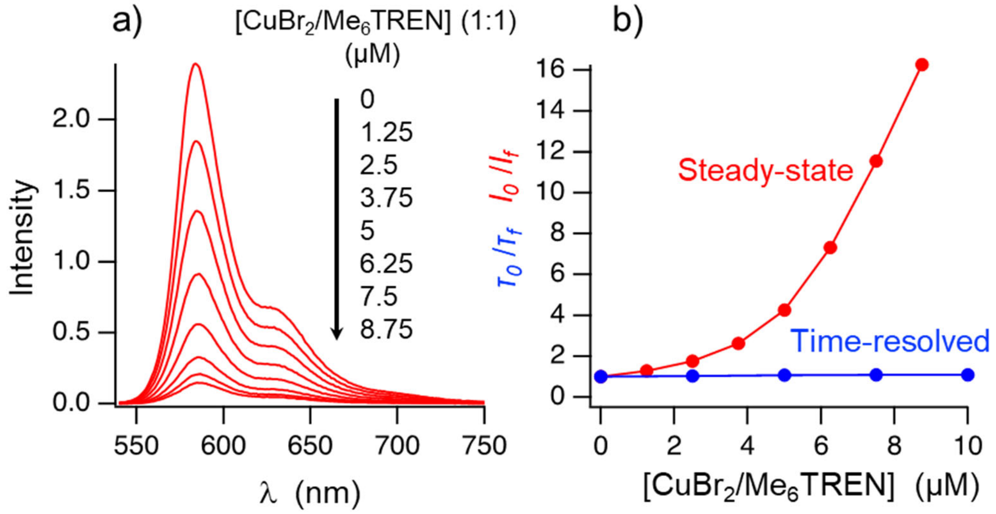

Figure S18: a) Fluorescence spectra of RB (6.5 μM) in DMSO in the absence and presence of varying concentrations of $CuBr_2$/Me_6TREN (1:1) (λ_{ex} = 520 nm). b) Stern-Volmer plots of steady-state fluorescence intensity (I_0 is the fluorescence in the absence of quencher and I_f is the fluorescence intensity in the presence of quencher) using data shown in a) (red) and Stern-Volmer plot of fluorescence lifetime measurements (λ_{ex} = 496 nm, pulsed LED; λ_{em} = 585 nm) determined by time-correlated single photon counting where τ_0 is the fluorescence in the absence of quencher and τ_f is the fluorescence intensity in the presence of quencher (blue).

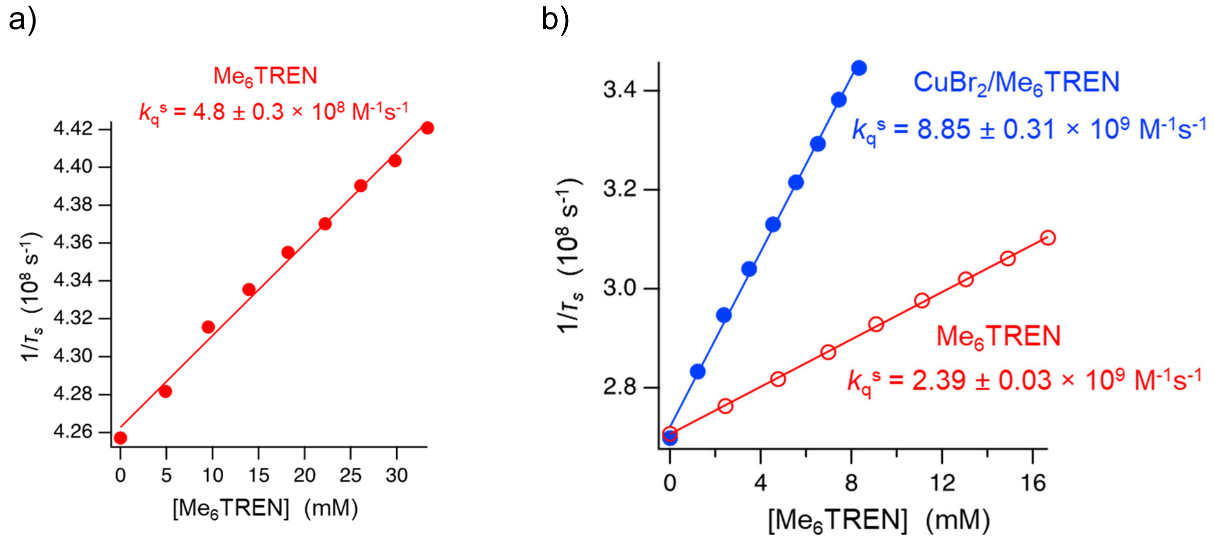

Figure S19 a) Determination of the bimolecular rate constants k_q^s of quenching of RB singlet excited states by Me_6TREN in MA/DMSO (1:1) using fluorescence lifetime measurements (λ_{ex} = 496 nm, pulsed LED). Inverse fluorescence lifetime of RB (λ_{em} = 585 nm) determined by time-correlated single photon counting vs. varying concentration of quencher, b) Determination of the bimolecular rate constants k_q^s of quenching of RD-6G singlet excited states by Me_6TREN (red) and $CuBr_2/Me_6TREN$ (1:1) (blue) in MA/DMSO (1:1) fluorescence lifetime measurements (λ_{ex} = 496 nm, pulsed LED). Inverse fluorescence lifetime of RD-6G (λ_{em} = 568 nm) determined by time-correlated single photon counting vs. varying concentration of quencher.
